# Vagal nerve stimulation induces vascular oscillations and enhances long-term learning

**DOI:** 10.1016/j.isci.2026.117413

**Published:** 2026-08-25

**Authors:** Junyu U. Chen, Yoko Ikoma, Ko Matsui

**Affiliations:** 1 Super-network Brain Physiology, Graduate School of Life Sciences, Tohoku University, Sendai 980-8577, Japan; 2 Super-network Brain Physiology, Graduate School of Medicine, Tohoku University, Sendai 980-8577, Japan

**Keywords:** vagus nerve stimulation, horizontal optokinetic responses, HOKR, learning and memory, vascular dynamics, brain metabolism

## Abstract

Despite its rapid emergence as a powerful approach for probing peripheral modulation of brain function, vagus nerve stimulation (VNS) is primarily understood through neuromodulatory pathways, leaving the potential contribution of vascular dynamics largely unexplored. Here, we examined whether VNS influences learning performance and its relationship with cerebrovascular dynamics using a chronic cuff-electrode system in freely moving mice. Using a cerebellum-dependent horizontal optokinetic response (HOKR) paradigm, we show that post-training VNS selectively enhances long-term learning. In parallel, *in vivo* fiber photometry reveals that VNS induces transient vascular fluctuations that evolve into oscillatory dynamics across repeated stimulation. The magnitude of these oscillations is increased by VNS and correlates with multi-day learning performance. These findings suggest that, in addition to neuromodulatory mechanisms, vascular dynamics may represent an underappreciated component of learning-related brain function. VNS-induced vascular oscillations are associated with multi-day learning, providing a new perspective on how peripheral signals influence central brain function.

## Highlights

• Vagus nerve stimulation enhances multi-day motor learning in mice
• VNS induces transient vascular volume dynamics in the cerebellar flocculus
• The magnitude of vascular oscillations is associated with learning performance
• VNS-enhanced learning may involve coordinated vascular and metabolic regulation

## Graphical abstract

**Figure:**
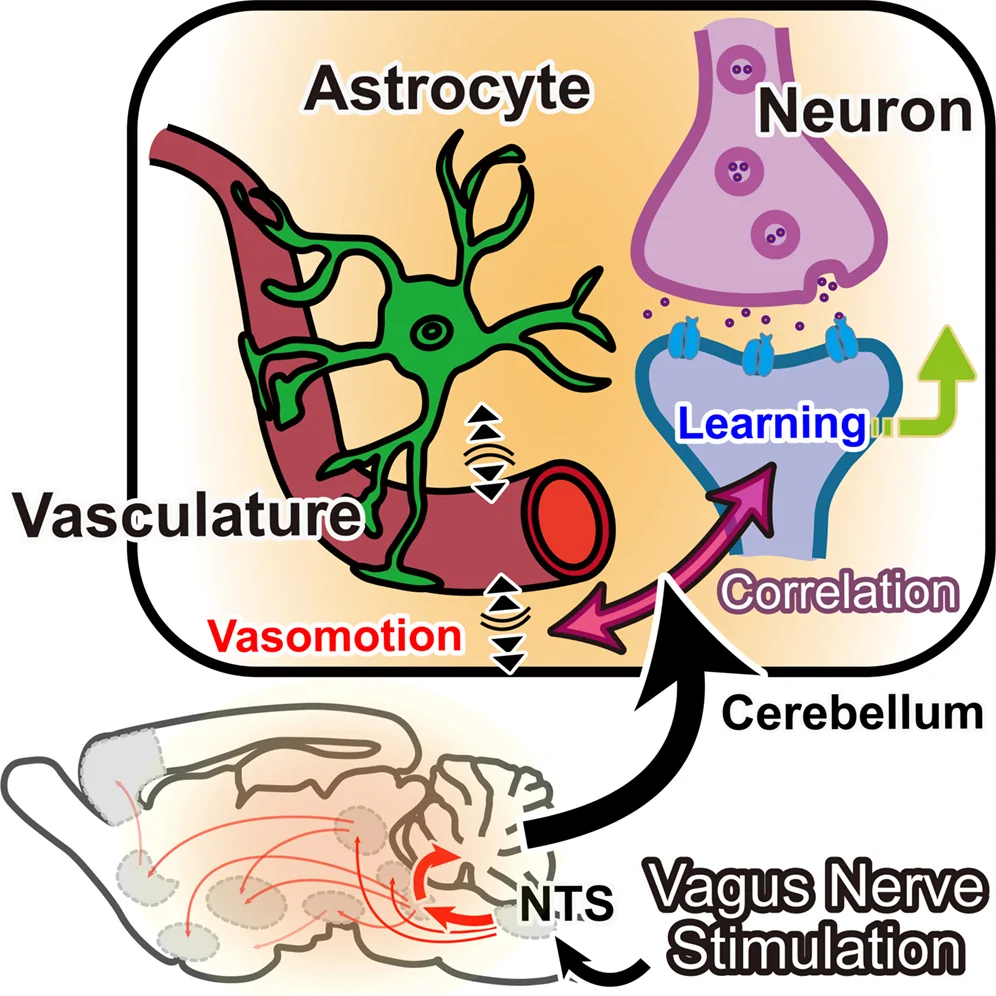

## Teaser

Biological sciences; Neuroscience; Techniques in neuroscience

## Introduction

The chemical, ionic, and molecular milieu of the brain critically shapes neuronal circuit function. Depending on the local brain environment, identical inputs can yield divergent outputs, and the likelihood of inducing synaptic plasticity (metaplasticity) can also be dynamically regulated,^1^^,^^2^ leading to marked variability in learning performance even under seemingly identical external conditions.

Such a local brain environment, including energy supply and metabolic support, is largely governed by non-neuronal elements such as glial cells and the vasculature.^3^^,^^4^ Learning and memory are expressed as changes in neuronal circuit performance, arising from activity-dependent modifications in synaptic efficacy. In particular, the induction and stabilization of long-term potentiation and depression (LTP/LTD), widely regarded as the core cellular mechanisms underlying learning,^5^^,^^6^^,^^7^ may be critically influenced by the local brain environment.^8^^,^^9^^,^^10^^,^^11^^,^^12^^,^^13^^,^^14^ Previous studies suggest that short-term learning primarily depends on transient synaptic changes, whereas long-term learning relies on protein synthesis and structural remodeling and is closely linked to energy metabolism.^7^^,^^15^^,^^16^ Within this framework, neurovascular coupling (NVC) regulates energy delivery to meet the metabolic demands of ongoing neuronal activity.^17^ Importantly, emerging evidence suggests that vascular dynamics and metabolic regulation may undergo coordinated adaptive changes and are associated with the induction of synaptic plasticity during learning.^18^

In recent years, the brain-body axis has emerged as a key regulator of brain function.^19^^,^^20^ Peripheral interoceptive signals have been shown to influence animal behavior.^21^ For example, cardiac- and gut-derived signals to the brain have been linked to anxiety- and depression-like states in rodents.^22^^,^^23^^,^^24^^,^^25^ These afferent signals predominantly converge onto the vagus nerve and are transmitted to the dorsal vagal complex, particularly the nucleus of the solitary tract (NTS), before being distributed across widespread brain regions in a bottom-up manner.^26^^,^^27^ This pathway engages multiple neuromodulatory systems, including cholinergic, noradrenergic, serotonergic, and dopaminergic signaling. Such vagus-mediated afferent activity may alter the local brain environment, thereby modulating neuronal circuit function and metaplasticity.

The vagus nerve can be electrically stimulated to artificially recruit a naturally existing peripheral-to-central pathway influencing brain function. Such vagus nerve stimulation (VNS) is an established clinical therapy for neurological disorders such as intractable epilepsy^28^; however, the precise physiological mechanisms underlying its effects in the brain remain incompletely understood. Nevertheless, given its therapeutic efficacy across various neurological conditions, VNS has increasingly been regarded as a unique experimental gateway for investigating peripheral modulation of central nervous system function.^29^ Yet, whether peripheral afferent signals, such as those evoked by VNS, are linked to parallel changes in vascular dynamics remains largely unknown.

Here, we developed a customized cuff electrode enabling chronic VNS in freely moving mice to directly test whether VNS-derived afferent signals influence learning performance and the local brain environment. Repeated training of the horizontal optokinetic response (HOKR) facilitates ocular motor performance through synaptic plasticity and refinement of neuronal circuitry in the cerebellum.^30^^,^^31^ The cerebellar flocculus is known to play a central role in this form of learning; however, whether long-term memory remains localized within the flocculus or is redistributed across brain regions over time remains debated.^32^^,^^33^^,^^34^ We found that delivering VNS immediately after HOKR training selectively enhanced long-term learning across days, suggesting a role in memory consolidation. Furthermore, *in vivo* fiber photometry combined with vascular imaging revealed that a single train of VNS induced transient vasoconstriction and vasodilation in local blood volume within the cerebellar flocculus. Because VNS trains were delivered every 40 s for a total of 20 min following HOKR training, repeated stimulation generated oscillatory vascular dynamics (hereafter referred to as vascular oscillations), which may be associated with enhanced blood flow and more efficient delivery of energy substrates. Notably, the magnitude of these vascular oscillations strongly correlated with the extent of long-term learning enhancement.

Based on these findings, we propose that peripheral afferent signals triggered by VNS are associated with changes in cerebrovascular dynamics and subsequent metabolic regulation, which may support long-term learning. However, a direct causal relationship between vascular oscillations and learning enhancement was not established in this study. These findings provide a framework for understanding how peripheral stimulation reshapes the brain environment and may guide the optimization of VNS-based therapeutic strategies.

## Results

### *In vivo* platform for vagal nerve stimulation in mice

**Figure 1:**
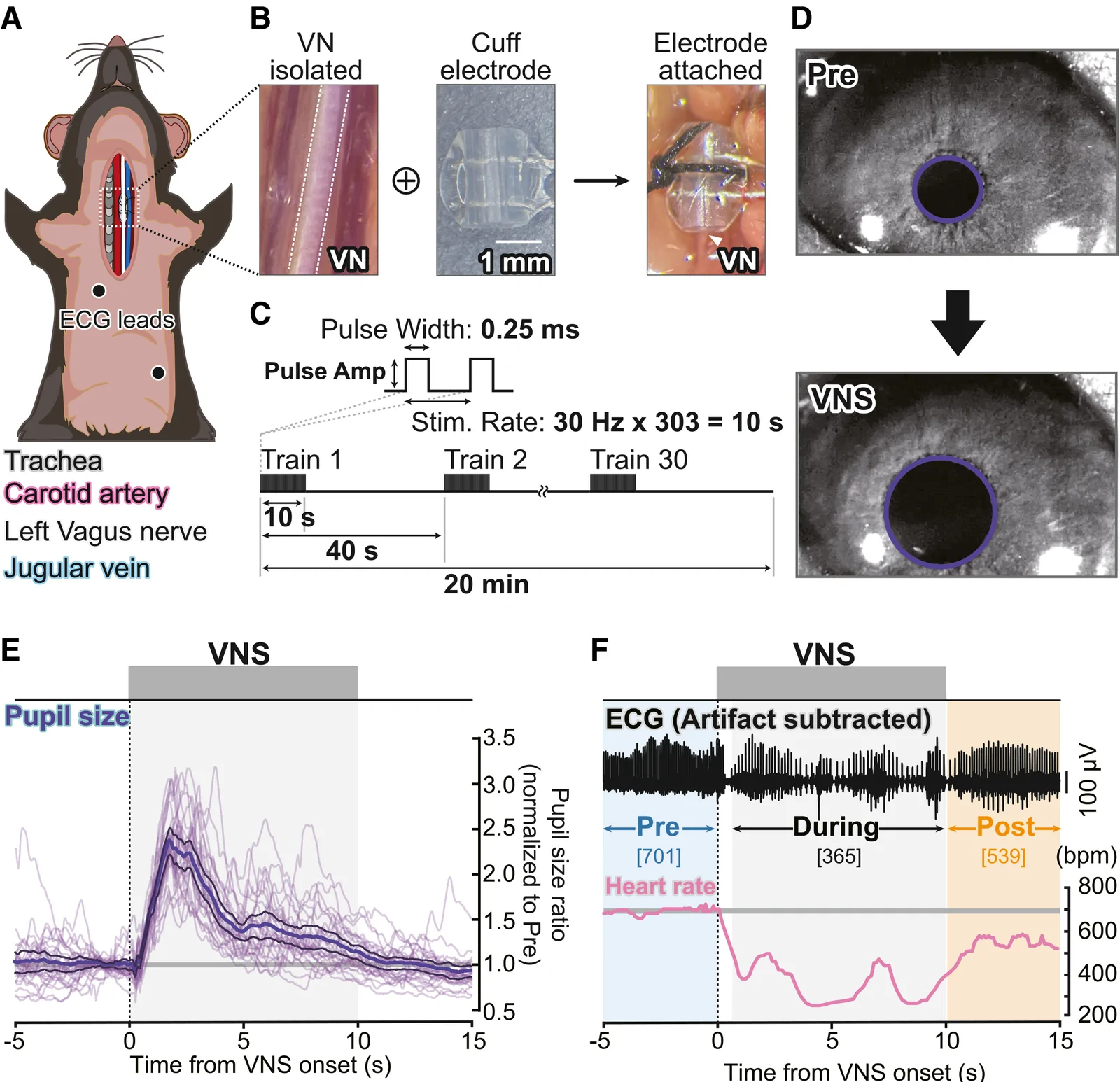
Surgical implantation and functional validation of a cervical VNS cuff electrode in mice (A) Schematic of cuff electrode implantation on the left cervical vagus nerve, showing surrounding anatomical structures (trachea, carotid artery, and jugular vein). ECG leads were implanted in the chest muscles for HR monitoring. (B) Representative images of the isolated vagus nerve and the implanted cuff electrode secured to the neck muscle. (C) Schematic of the VNS stimulation protocol. (D) Representative images of VNS-evoked pupil dilation (blue circles indicate estimated pupil boundaries). (E) Representative pupil size traces showing transient dilation following VNS onset, peaking during stimulation. Individual trials are shown in light blue-purple, with the mean trace (dark line) and confidence interval (±2 SEM). (F) Representative ECG trace showing a transient reduction in HR during VNS.

To investigate the role of VNS in regulating brain function, we targeted the left cervical vagus nerve and developed a customized cuff electrode that enabled controlled stimulation in freely moving mice over extended periods (Figures 1A and 1B). Because the physiological effects of VNS can vary depending on the experimental species, cuff-electrode design, electrode properties, and the specific combination of stimulation parameters, we selected the stimulation protocol based on parameter ranges commonly used in previous rodent VNS studies and our preliminary optimization.^28^^,^^35^^,^^36^^,^^37^ Trains of monophasic constant-current pulses (1.5 mA for the representative stimulation protocol shown in Figure 1C; 250 μs pulse width) were delivered at 30 Hz for 10 s, repeated 30 times at 40-s intervals (Figure 1C). The functionality of the implanted electrodes was validated by VNS-evoked pupil dilation and heart rate reduction, confirming effective vagal activation.

VNS-evoked pupil dilation has been previously reported as a physiological signature of vagal activation.^37^ In the present study, VNS delivered through the implanted cuff electrode reliably evoked transient pupil dilation during stimulation (Figures 1D and 1E). When VNS trains were repeatedly delivered at 40-s intervals, the normalized amplitude of pupil dilation remained stable across trials. No significant difference was observed between the mean normalized amplitudes of the first five and last five trains (t_(7)_ = 0.4325, *p* = *0*.*68*, n.s., Figures S1A–S1D). We next quantified the temporal characteristics of VNS-evoked pupil dilation. The latency to peak dilation (3.28 ± 0.38 s) and latency to peak acceleration (499.56 ± 21.24 ms) were consistent with previous reports^38^^,^^39^ (Figures S1E and S1F). In addition, baseline pupil size remained stable following repeated VNS, with no significant drift detected (t_(7)_ = 2.309, *p* = *0*.*08*, n.s., Figure S1G).

VNS-induced heart rate (HR) reduction has also been reported as a peripheral physiological signature of vagal activation.^40^^,^^41^ In the present study, VNS induced a transient reduction in HR during the stimulation period, followed by a sustained effect after stimulation (Figure 1F). Absolute HR values were expressed as beats per minute (bpm), whereas normalized HR changes were expressed as HR ratio relative to the pre-VNS baseline. Because stimulation artifacts interfered with ECG recordings, artifact-subtracted ECG traces were obtained using custom subtraction and HR detection algorithms (Figure S2). The first VNS train induced a significant reduction in normalized HR ratio (pre vs. during VNS: 1.000 vs. 0.7085 ± 0.0410, mean ± SEM; *p* < *0*.*001*). Across repeated VNS trains, a significant difference was observed between the mean HR responses of the first five and last five trains (t_(5)_ = 4.625, *p* = *0*.*006*, Figures S3A–S3E). In contrast to the absence of baseline drift in pupil size, baseline HR exhibited a significant drift following repeated VNS (t_(11)_ = 7.620, *p* < *0*.*001*). Moreover, baseline HR was negatively correlated with the corresponding HR ratio (r = −0.45, *p* < *0*.*001*, Figures S3F and S3G).

### Vagus nerve stimulation facilitates long-term learning performance

Using this *in vivo* VNS platform, we next examined whether VNS modulates learning performance. Based on previous reports that cortical plasticity depends on VNS intensity and that pupil size reflects neuromodulatory activity in the central brain,^42^^,^^43^ we first evaluated the relationship between VNS intensity and pupil dilation. Increasing VNS intensity elicited progressively larger pupil dilation. Previous studies have suggested that altering stimulation polarity can preferentially engage afferent or efferent vagal fibers, thereby enhancing efficacy while minimizing off-target effects.^44^ However, in contrast to these reports, stimulation polarity did not significantly affect pupil dilation across graded stimulation intensities (Figures S4A–S4D). Based on these results and previous findings, moderate-to-high VNS intensities (0.6 and 1.5 mA) with a cathode-cephalad configuration were used for subsequent experiments.

**Figure 2:**
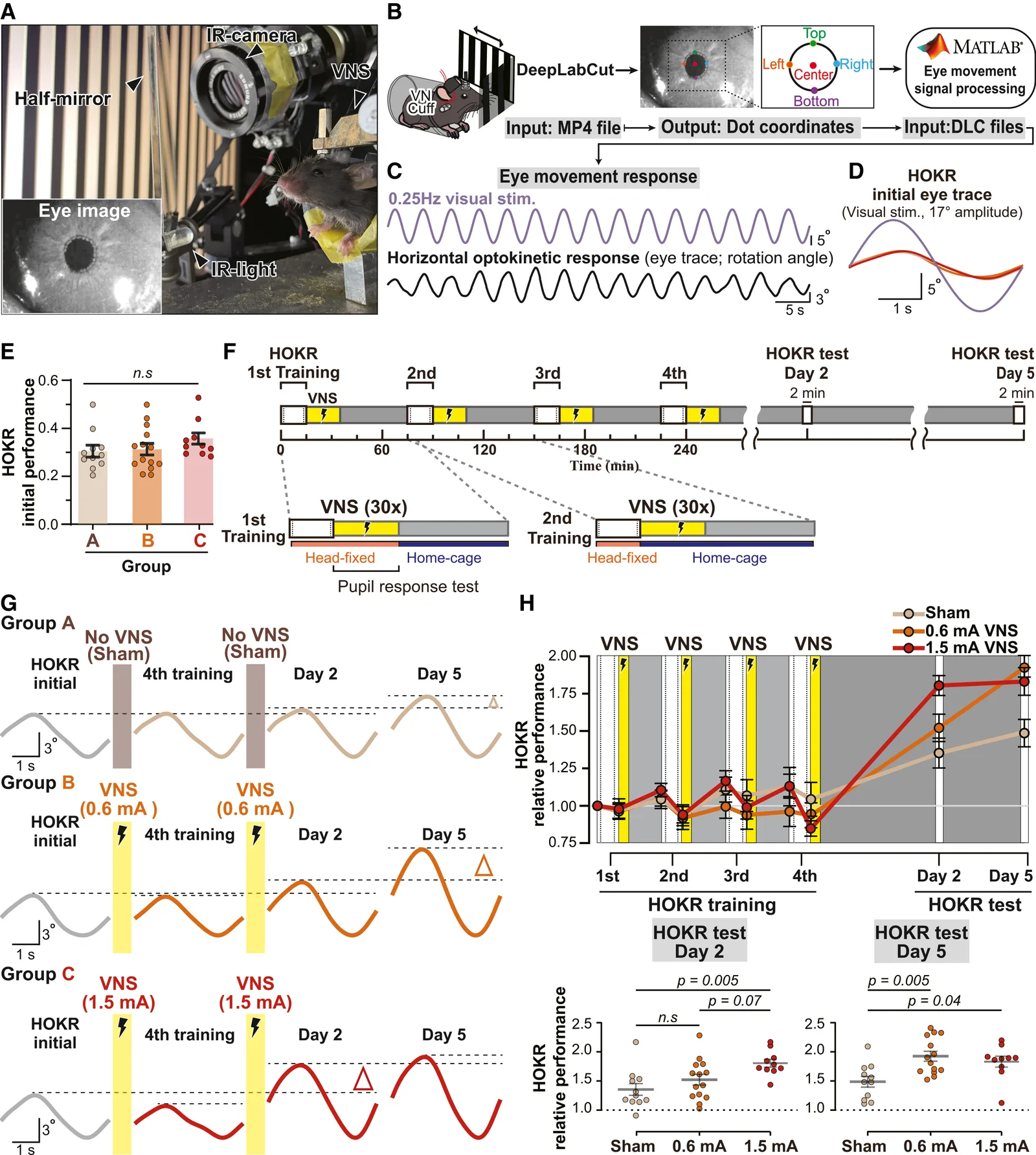
VNS induces delayed enhancement of long-term learning performance (A) Schematic of the eye movement-tracking setup. The mouse eye was recorded using an infrared (IR) camera (inset), with the head fixed in front of two perpendicular monitors. (B) Pupil position tracking using DeepLabCut, with extracted eye movement coordinates. (C) Visual stimulus consisting of horizontally oscillating vertical stripes (0.25 Hz) and corresponding horizontal optokinetic response (HOKR) traces. (D) Representative averaged eye movement traces during the initial 3 min. Visual stimulus (mauve, 17° amplitude) and experimental groups (group A, dark brown; group B, dark orange; group C, crimson) are shown. (E) Initial HOKR performance did not differ significantly among groups (one-way ANOVA, F_(2, 32)_ = 1.231, *p* = 0.31). Sample sizes: *n* = 11 (group A), *n* = 14 (group B), and *n* = 10 (group C). (F) Schematic of the spaced HOKR training paradigm and timing of VNS. Four 15-min training sessions were performed at 1-h intervals. Probe tests were conducted on days 2 and 5. VNS was delivered after each training session; during the first session, mice remained head-fixed for validation, whereas subsequent VNS was delivered in the home cage. (G) Representative averaged eye traces from each group at the start of training, after the fourth session (day 1), and during probe tests on days 2 and 5. (H) VNS selectively enhanced late-offline learning. No significant differences were observed immediately after training on day 1. In contrast, 1.5 mA VNS significantly increased HOKR performance compared with the sham condition on day 2 (Tukey-adjusted *p* = *0*.*005*), whereas 0.6 mA VNS had no significant effect (adjusted *p* = *0*.*46*). On day 5, both the 0.6 mA and 1.5 mA VNS groups showed significantly greater HOKR performance than the sham group (adjusted *p* = *0*.*005* and *p* = *0*.*04*, respectively). A mixed-effects model fitted by restricted maximum likelihood (REML), with fixed effects assessed using type III tests, revealed a significant interaction between training session and VNS condition (F_(18, 288)_ = 4.625, *p* < *0*.*001*). Post hoc comparisons were performed using Tukey’s multiple-comparisons test. Complete statistical results, including mean differences and 95% confidence intervals, are provided in the Data S1. Data are presented as mean ± SEM (sham, *n* = 11; 0.6 mA VNS, *n* = 14; 1.5 mA VNS, *n* = 10). n.s., not statistically significant.

We next employed a cerebellum-dependent horizontal optokinetic response (HOKR) motor learning paradigm to examine whether VNS facilitates learning.^30^ Visual presentation of horizontally oscillating vertical stripes elicited HOKR in head-fixed mice, and eye movements were recorded and analyzed to obtain eye rotation angle traces (Figures 2A–2C). At the beginning of training, eye movements were poorly matched to the visual stimulus, resulting in smaller eye rotation amplitudes (Figure 2D). Initial HOKR performance was defined as the ratio of eye movement amplitude to stimulus amplitude during the first 3 min of the first training session. At this stage, all mice underwent identical experimental procedures prior to group assignment. No significant differences in initial HOKR performance were observed among animals that were later assigned to the three experimental groups (no VNS, 0.6 mA VNS, and 1.5 mA VNS; ordinary one-way ANOVA, F_(2, 32)_ = 1.231, *p* = *0*.*31*, n.s., Figure 2E). This confirms that all groups started from comparable baseline performance.

In the spaced training paradigm, mice underwent four training sessions (15 min each) on day 1 at 1-h intervals, as described previously.^18^^,^^31^ Within-session increases in eye movement amplitude were defined as online learning, whereas gains between sessions were defined as offline learning. Gains from the fourth training session to probe tests conducted on days 2 and 5 were defined as late-offline learning.

Following each training session, VNS was delivered immediately in groups B (0.6 mA) and C (1.5 mA), whereas no stimulation was applied in group A (sham). All mice remained head-fixed after the first training session for a comparable duration. In VNS groups (groups B and C), pupil dilation was monitored to confirm effective stimulation (Figure 2F), and mice failing validation criteria were excluded (see supplemental information). In sham animals, although no stimulation was delivered during HOKR training paradigm, cuff electrode functionality was confirmed after the day 5 test via VNS-evoked pupil dilation, and animals failing validation were excluded. After the second, third, and fourth training sessions, mice were released from head fixation immediately following HOKR training, and in the VNS groups, VNS was delivered immediately thereafter while the animals were freely moving.

A mixed-effects model (REML), with fixed effects assessed using type III tests, revealed a significant interaction between training session and VNS condition (F_(18, 288)_ = 4.625, *p* < *0*.*001*; Figure 2H). No significant learning gain was observed in the sham group on day 1, either during online or offline phases. However, late-offline learning emerged on days 2 and 5, although not fully consistent with previous reports.^18^^,^^31^ A similar lack of effect was observed in VNS groups on day 1, indicating that VNS did not influence online or early offline learning. In contrast, post hoc Tukey’s multiple-comparisons test showed that VNS selectively enhanced late-offline HOKR learning in an intensity-dependent temporal profile, with significant improvement on day 2 in group C (sham vs. group C: 1.355 ± 0.102 vs. 1.805 ± 0.068, adjusted *p* = *0*.*005*) and on day 5 in both groups B and C (sham vs. group B: 1.487 ± 0.095 vs. 1.925 ± 0.076, adjusted *p* = *0*.*005*; sham vs. group C: 1.487 ± 0.095 vs. 1.832 ± 0.103, adjusted *p* = *0*.*04*). Values are presented as mean ± SEM.

### VNS elicits cerebellar vascular volume dynamics in the cerebellar flocculus

**Figure 3:**
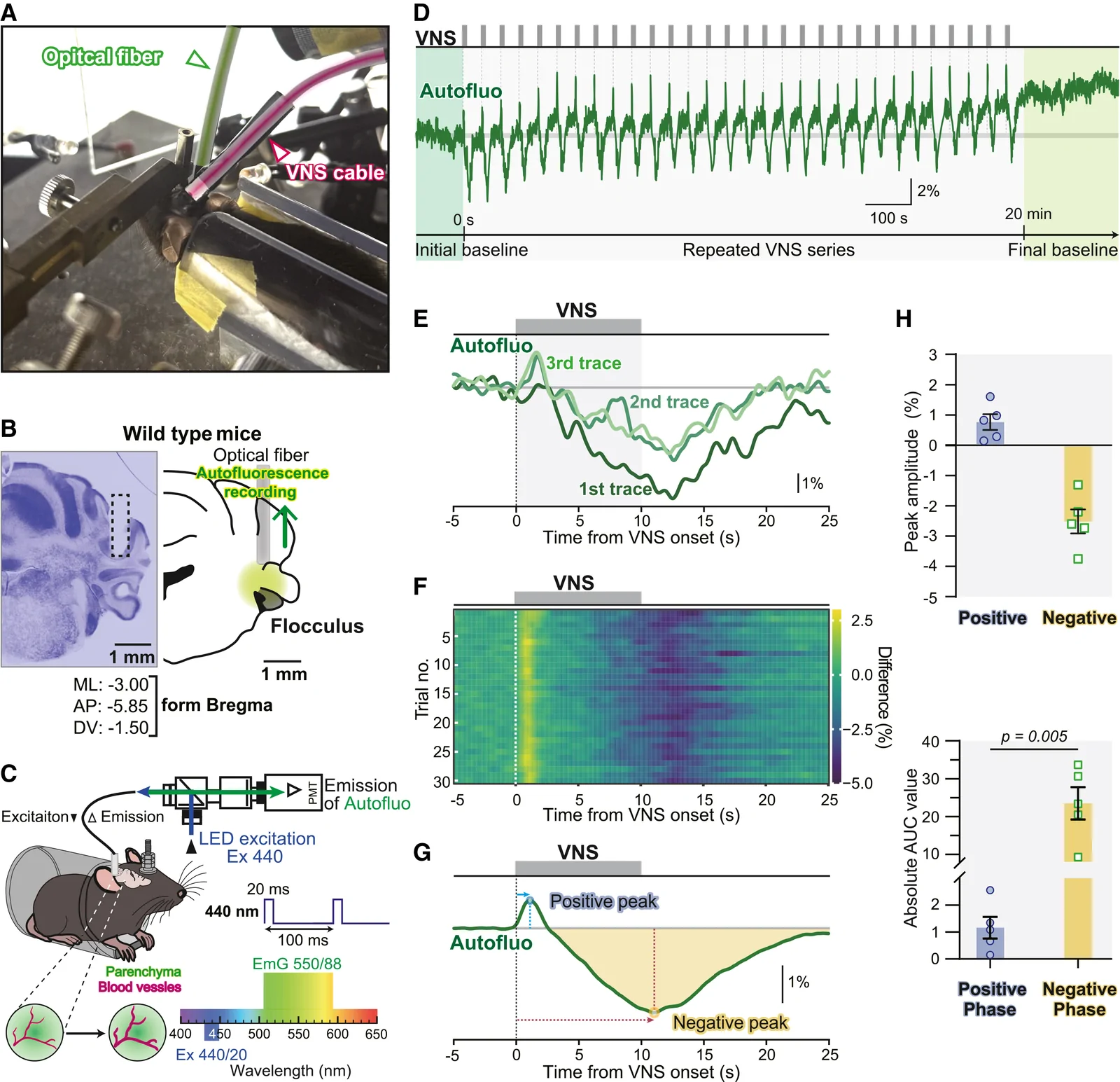
VNS induces biphasic autofluorescence fluctuations in the cerebellar flocculus (A) Experimental setup showing an optical fiber implanted near the cerebellar flocculus and connected to the fiber photometry system (green). VNS cuff electrode wires were connected to a constant-current stimulator (red). (B) Image and schematic of optical fiber placement relative to the cerebellar flocculus. (C) Schematic of the autofluorescence photometry configuration. Excitation light (440 nm) was delivered through the optical fiber, and emitted autofluorescence was collected through the same fiber and detected by a photomultiplier tube (PMT). (D) Representative autofluorescence trace during VNS. (E) Autofluorescence responses evoked by the first to third VNS trials. (F) Trial-to-trial autofluorescence responses across a VNS series in an example mouse. (G) Average autofluorescence trace across the VNS series, showing biphasic dynamics with an initial positive deflection followed by a delayed negative deflection. (H) Quantification of positive and negative peak amplitudes (top) and absolute AUC values (bottom) of VNS-evoked autofluorescence signals. The negative phase exhibited a significantly larger absolute AUC than the positive phase (paired two-tailed *t* test, t_(4)_ = 5.698, *p* = 0.005). Each data point represents one animal (*n* = 5), averaged across three episodes (30 VNS trials per episode). Data are presented as mean ± SEM. VNS onset was defined as 0 s. Signals were low-pass filtered at 1 Hz.

To examine vascular dynamics during VNS, we visualized brain blood volume (BBV) fluctuations in the cerebellar flocculus, a region essential for HOKR learning.^30^^,^^34^ Autofluorescence-based “shadow” imaging was employed, in which signals are detected in the brain parenchyma but are reduced within blood vessels due to hemoglobin absorption^18^ (Figures 3A–3C). This approach has been previously validated using fiber photometry.^10^^,^^11^ Using this method, individual VNS trains evoked transient fluctuations in autofluorescence intensity near the tip of the optical fiber implanted in the cerebellar flocculus. These responses were highly reproducible across repeated stimulations (Figures 3D–3F). Individual responses exhibited biphasic dynamics, consisting of a rapid positive deflection followed by a delayed negative deflection (Figure 3G; Figure S5B). Quantitative analysis revealed that the magnitude of the negative deflection was significantly greater than that of the positive deflection (absolute AUC: 23.486 ± 4.287 vs. 1.163 ± 0.410, mean ± SEM; t_(4)_ = 5.698, *p* = *0*.*005*; Figure 3H).

To further characterize these responses, we analyzed trial-by-trial dynamics across repeated VNS trains (Figures S5A–S5D). The largest peak amplitude of the negative deflection was observed during the initial VNS trains; however, no significant difference was detected between the mean amplitudes of the first five and last five trains (t_(4)_ = 1.733, *p* = *0*.*16*, n.s.). In contrast, the peak latency of the negative deflection was significantly prolonged across repeated stimulation (t_(4)_ = 3.176, *p* = *0*.*03*; Figure S5E). Furthermore, across mice at the same stimulation intensity, the magnitude of the negative deflection was negatively correlated with peak latency (r = −0.54, *p* = *0*.*02*, Figure S5F).

### Direct visualization of VNS-elicited cerebellar vascular volume dynamics

**Figure 4:**
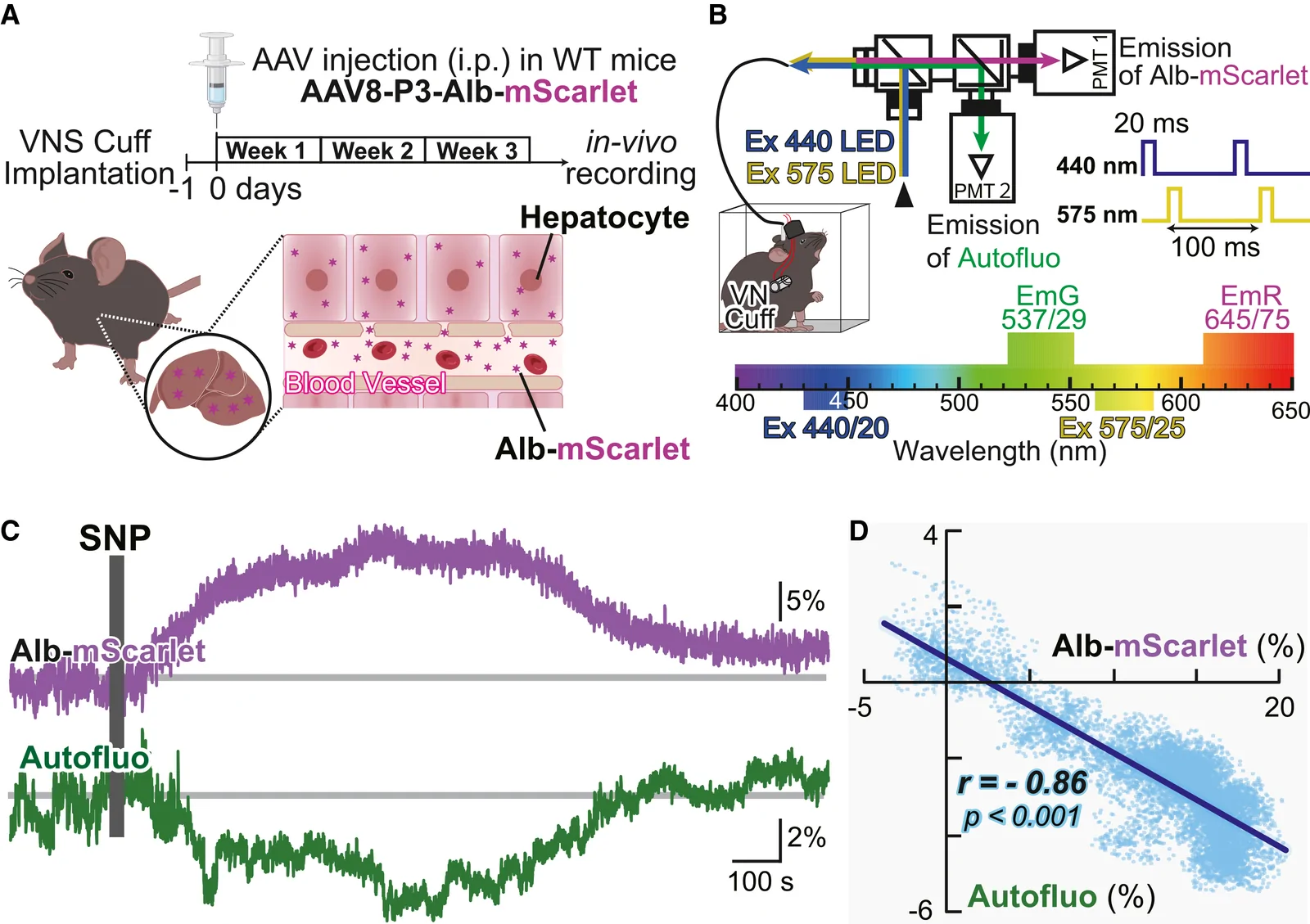
Reciprocal changes in Alb-mScarlet and autofluorescence signals during SNP-induced vasodilation (A) Experimental timeline showing VNS cuff electrode implantation (day −1), AAV injection (day 0), and a 3-week expression period for Alb-mScarlet (top). Schematic of Alb-mScarlet expression in hepatocytes and secretion into the bloodstream (bottom). (B) Schematic of the dual-channel fiber photometry system for simultaneous detection of autofluorescence and Alb-mScarlet signals. Excitation light (440 nm and 575 nm) was delivered sequentially, and emitted fluorescence was collected through the same optical fiber, spectrally separated, and detected by photomultiplier tubes (PMTs). (C) Representative fluorescence traces during SNP-induced vasodilation, showing an increase in Alb-mScarlet signal accompanied by a decrease in autofluorescence signal, followed by a gradual return to baseline. (D) Scatterplot of Alb-mScarlet and autofluorescence signals corresponding to the period shown in (C). A strong negative correlation was observed (*n* = 10,018 data points, r = −0.86, *p* < 0.001, linear regression), indicating that autofluorescence signals can serve as an indirect proxy for fluctuations in BBV. Signals were low-pass filtered at 1 Hz.

To directly visualize vascular volume dynamics, we expressed an albumin-mScarlet (Alb-mScarlet) fusion protein in the liver via adeno-associated viral transduction. The fusion protein was secreted into the bloodstream and circulated systemically, enabling fluorescence-based visualization of vascular volume^45^^,^^46^ (Figure 4A). A dual-fluorescence recording system was established to simultaneously monitor autofluorescence and Alb-mScarlet signals using sequential excitation at 440 nm and 575 nm, respectively (Figure 4B). Following subcutaneous injection of the vasodilator sodium nitroprusside (SNP), Alb-mScarlet fluorescence increased, whereas autofluorescence decreased, showing a strong inverse relationship between the two signals (r = −0.86, *p* < *0*.*001*; Figures 4C and 4D).

**Figure 5:**
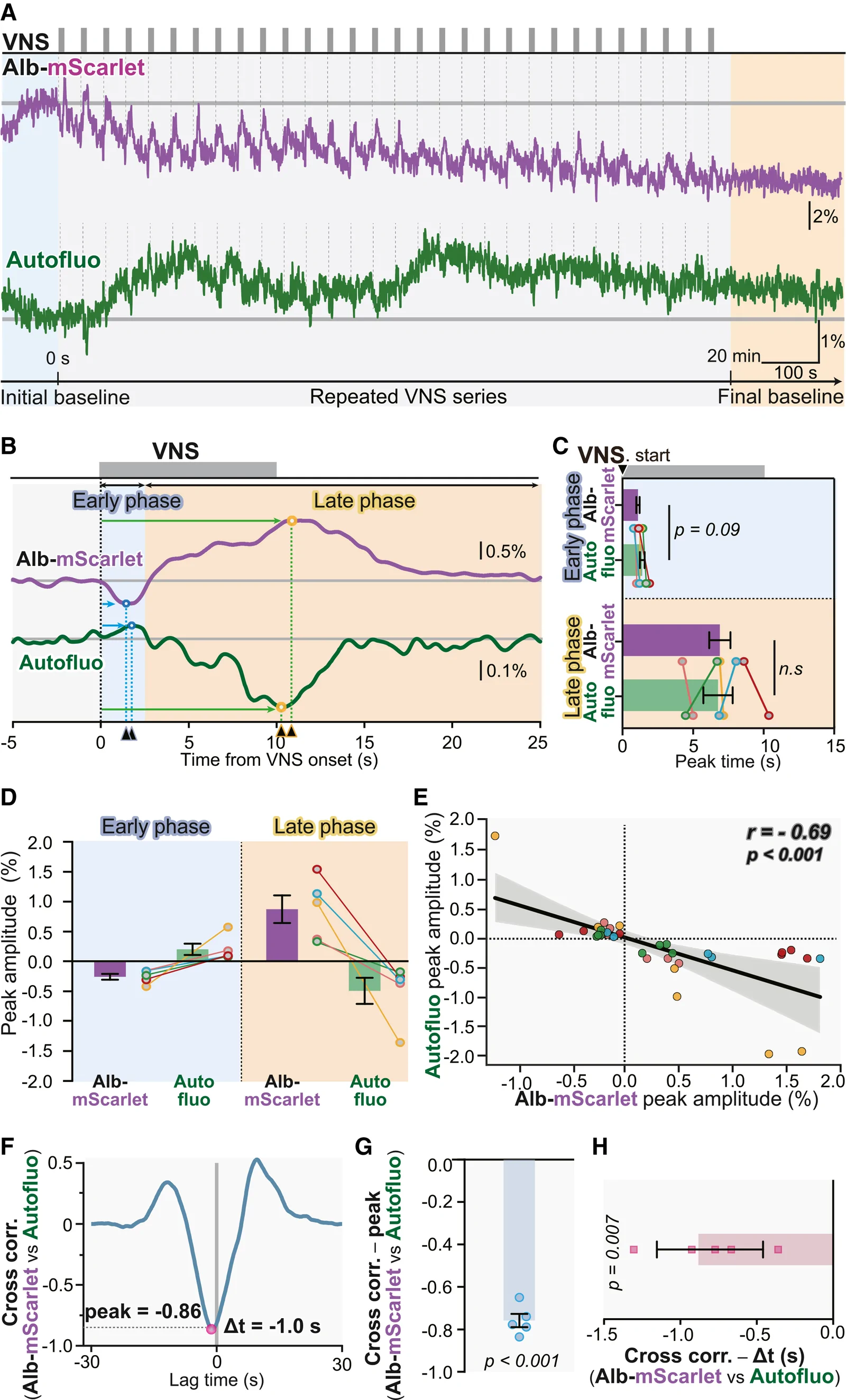
Reciprocal biphasic fluctuations of Alb-mScarlet and autofluorescence signals during VNS (A) Simultaneous Alb-mScarlet and autofluorescence traces showing transient reciprocal fluctuations evoked by individual VNS trials across a stimulation series. (B) Average traces reveal two sequential response phases (early and late). In the early phase, Alb-mScarlet exhibited a negative deflection and autofluorescence a positive deflection, whereas in the late phase, Alb-mScarlet showed a positive deflection and autofluorescence a negative deflection. (C) Peak times of Alb-mScarlet and autofluorescence signals did not differ significantly in either phase (paired two-tailed *t* test; early phase: t_(4)_ = 2.251, *p* = 0.09, n.s.; late phase: t_(4)_ = 0.181, *p* = 0.87, n.s.). (D) Peak amplitudes of Alb-mScarlet and autofluorescence signals in the early and late phases. (E) Scatterplot of peak amplitudes from both phases showing a significant negative correlation (r = −0.69, *p* < 0.001, linear regression). Each data point represents one phase from a single episode (30 VNS trials; 36 episodes from 5 mice). (F) Cross-correlation between Alb-mScarlet and autofluorescence signals, showing a prominent negative peak at a lag of approximately −1.0 s. (G) Negative peak values of cross-correlation across animals (one-sample *t* test, t_(4)_ = 24.38, *p* < 0.001). (H) Lag time of Alb-mScarlet relative to autofluorescence signals (one-sample *t* test, t_(4)_ = 5.195, *p* = 0.007). Except for (E), each data point represents one animal (*n* = 5), averaged across three or four independent VNS series (30 trials per series). Data are presented as mean ± SEM. VNS onset was defined as 0 s. Signals were low-pass filtered at 1 Hz.

During VNS, each trial elicited biphasic fluctuations in Alb-mScarlet fluorescence, consisting of an initial negative deflection followed by a delayed positive deflection. These dynamics were largely reciprocal to autofluorescence signals but not perfectly mirrored, with slight temporal offsets observed between corresponding peaks during both early and late phases of VNS delivery (early phase: 1.089 ± 0.117 vs. 1.392 ± 0.164 s, t_(4)_ = 2.25, *p* = *0*.*09*; late phase: 6.891 ± 0.721 vs. 6.762 ± 1.049 s, t_(4)_ = 0.18, *p* = *0*.*87*; Figures 5A–5C). Consistent amplitude relationships were observed between Alb-mScarlet and autofluorescence signals, resulting in a significant negative correlation between their peak amplitudes (r = −0.69, *p* < *0*.*001*; Figures 5D and 5E). Increasing VNS intensity produced larger deflections in both signals, with a saturation effect at higher intensities. In addition, cathode-cephalad stimulation produced larger deflections than cathode-caudad stimulation (Figure S6).

Cross-correlation analysis revealed a prominent negative peak between Alb-mScarlet and autofluorescence signals (representative r = −0.86) with a lag of approximately −1.0 s (Figure 5F). Across mice, this relationship was consistent (peak r = −0.76 ± 0.031, *p* < *0*.*001*; lag time = −0.81 ± 0.153 s, *p* = *0*.*007*; Figures 5G and 5H). Trial-by-trial analysis demonstrated stable cross-correlation features across repeated VNS trains, with no significant differences between early and late trials (Figure S7).

Baseline analysis revealed a significant cumulative increase in autofluorescence following repeated stimulation (3.474 ± 0.287, t_(4)_ = 12.10, *p* < *0*.*001*), whereas Alb-mScarlet fluorescence showed no significant change (−1.230 ± 1.701, t_(4)_ = 0.720, *p* = *0*.*51*; Figures S8A–S8D). Accordingly, no significant correlation was observed between baseline drifts of the two signals (r = −0.36, *p* = *0*.*14*; Figure S8E).

These results demonstrate that VNS induces coordinated and temporally structured vascular volume dynamics in the cerebellum. Notably, the subtle differences between Alb-mScarlet and autofluorescence signals, including temporal delays and baseline drifts, may reflect additional contributions from concentration changes in metabolic-related autofluorescence components, such as flavins, beyond vascular shadowing effects.

### Post-training vascular volume oscillations are associated with long-term HOKR learning performance

**Figure 6:**
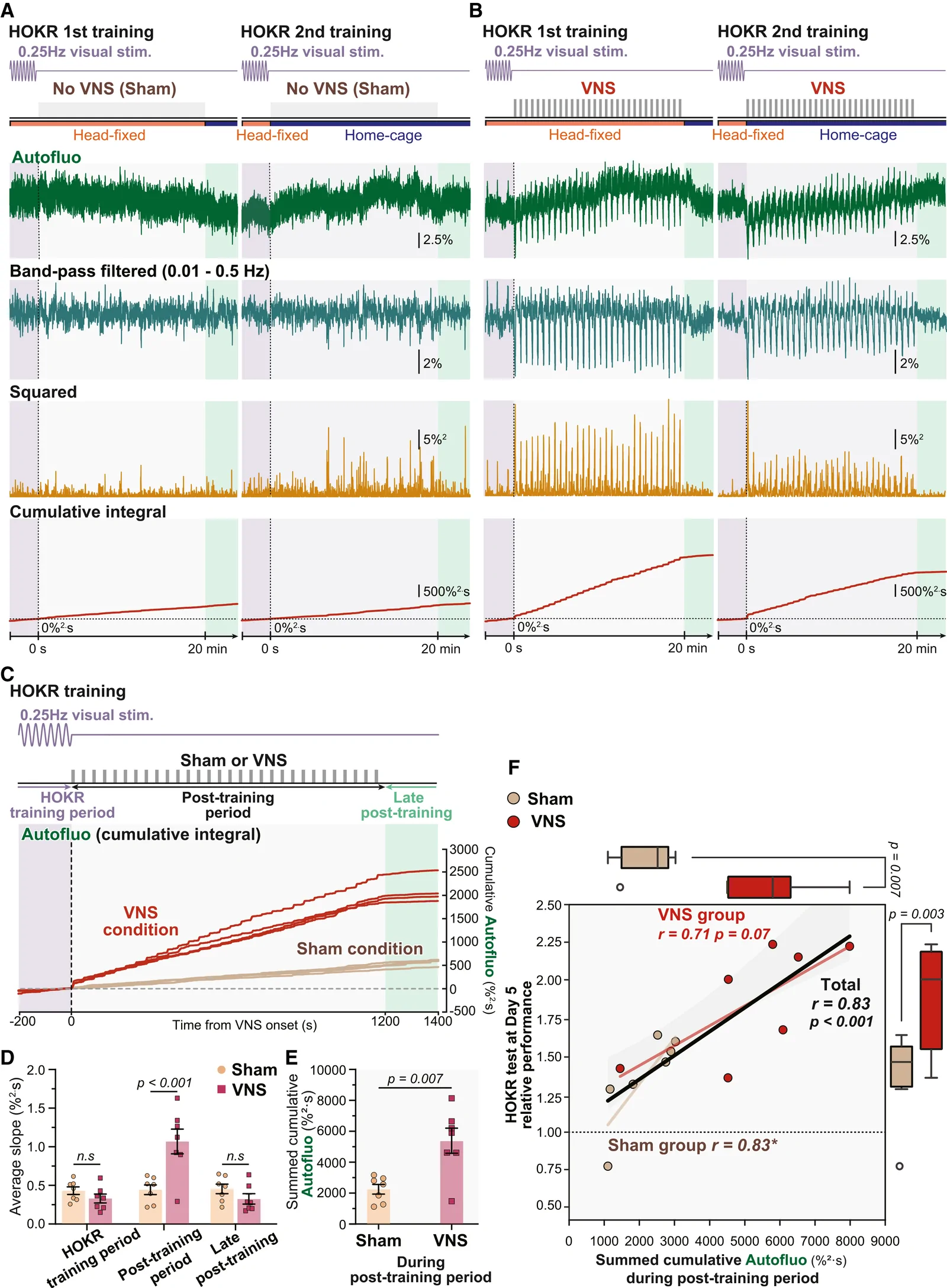
VNS enhances cumulative vascular oscillations and is associated with improved long-term HOKR learning (A) Representative autofluorescence traces during the first and second training sessions under the sham condition. Raw traces (top) were band-pass filtered (0.01–0.5 Hz), squared, and cumulatively integrated (bottom). Post-training onset was defined as 0 s; signals from −20 to 0 s were used as baseline and normalized to zero. (B) Representative autofluorescence traces during the first and second training sessions under the VNS condition, processed as in (A). (C) Cumulative integral of autofluorescence signals across the first to fourth training sessions under sham and VNS conditions. (D) Average slope of autofluorescence signals across defined time periods. Two-way repeated-measures ANOVA revealed a significant interaction between time period and VNS condition (F_(2, 24)_ = 18.55, *p* < 0.001). Bonferroni post hoc tests showed no difference during the training period (*p* > 0.99, n.s.) or late post-training period (*p* = 0.86, n.s.), but a significant increase during the post-training period (*p* < 0.001). (E) Cumulative integral of autofluorescence signals summed across sessions. VNS significantly increased cumulative signal magnitude compared with sham (Welch’s *t* test, t_(7.83)_ = 3.65, *p* = 0.007). (F) Relationship between cumulative autofluorescence and HOKR performance on day 5. A strong positive correlation was observed when data were pooled across conditions (r = 0.88, *p* < 0.001, *n* = 14). When analyzed separately, a significant correlation was observed in the sham group (r = 0.83, *p* = 0.04, *n* = 7), whereas a similar positive trend was observed in the VNS group (r = 0.71, *p* = 0.07 n.s., *n* = 7). HOKR performance on day 5 was higher in the VNS group (1.5 mA) compared with sham (see Data S1). Data are presented as mean ± SEM.

To quantify vascular volume oscillations following HOKR training, autofluorescence intensity traces recorded near the cerebellar flocculus were band-pass filtered (0.01–0.5 Hz), squared, and cumulatively integrated to estimate the magnitude of vascular oscillations (Figures 6A and 6B). Autofluorescence fluctuations were observed under both sham and VNS conditions; however, repeated VNS induced a steeper increase in these fluctuations, resulting in a greater overall magnitude of oscillatory activity (Figure 6C). The average slope during the post-training period was significantly higher in the VNS group than in the sham group (1.067 ± 0.161 vs. 0.442 ± 0.063, mean ± SEM; *p* < *0*.*001*). In contrast, no significant difference was observed between groups during the late post-training period (0.453 ± 0.062 vs. 0.322 ± 0.066, mean ± SEM; *p* = *0*.*86*; Figure 6D). Consistently, the cumulative magnitude of vascular oscillations across the first to fourth post-training sessions was significantly greater in the VNS group than in the sham group (5,372.69 ± 795.6 vs. 2,248.73 ± 314.2, mean ± SEM; t_(7.83)_ = 3.65, *p* = *0*.*007*; Figure 6E).

To assess the relationship between vascular oscillations and learning performance, the cumulative oscillation magnitude was plotted against HOKR performance on day 5. A significant positive correlation was observed in the sham group (r = 0.83, *p* = *0*.*04*), suggesting that naturally occurring vascular oscillations may be associated with long-term plasticity. A positive trend was also observed in the VNS group (r = 0.71, *p* = *0*.*07*), and when data from both groups were pooled, a stronger positive correlation emerged (r = 0.88, *p* < *0*.*001*; Figure 6F). Consistent with these findings, HOKR performance on day 5 was significantly higher in the VNS group (1.5 mA) than in the sham group (1.392 ± 0.117 vs. 1.866 ± 0.128, mean ± SEM; *p* = *0*.*003*). These results indicate that the magnitude of vascular oscillations, including those enhanced by VNS, is closely associated with long-term learning performance.

## Discussion

Our results demonstrate that VNS selectively enhances long-term learning performance, with a delayed time course consistent with, but not establishing, a contribution of memory consolidation-related processes. Importantly, VNS also induced coordinated and temporally structured vascular volume dynamics in the cerebellum, and the magnitude of these vascular oscillations was strongly associated with long-term learning performance. Although this relationship is correlational and does not establish causality, it suggests that vascular dynamics may represent an additional mechanism supporting memory consolidation, potentially by facilitating sustained energy supply. These findings position vascular dynamics not merely as a downstream consequence of neuronal activity, but as a parallel regulatory axis that may shape the conditions under which long-term plasticity emerges (Figure S9).

In contrast to previous studies, HOKR training did not produce robust online or early offline learning in the control group.^18^^,^^31^ This discrepancy may reflect differences in experimental conditions, including surgical procedures and extended head-fixation duration after the first HOKR training session in both control and VNS stimulated groups, although the precise cause remains unclear.

Previous studies have shown that VNS can enhance learning performance, including perceptual and category learning, facilitate memory extinction, and alleviate depression-like behaviors.^47^^,^^48^^,^^49^^,^^50^ However, findings remain inconsistent. Some studies report immediate behavioral improvements following acute VNS,^35^^,^^51^ whereas others describe delayed effects emerging after repeated stimulation or on subsequent days, consistent with our observations.^48^^,^^52^ In contrast, several studies have reported no significant facilitation of learning.^53^ These discrepancies suggest that VNS effects depend strongly on stimulation parameters such as timing, intensity, and frequency.^28^

In the present study, post-training VNS significantly enhanced late-offline learning on days 2 and 5. Stimulation intensity influenced the temporal profile of this effect: higher intensity (1.5 mA) produced earlier facilitation, whereas lower intensity (0.6 mA) required more time to emerge. However, the magnitude of enhancement was comparable by day 5, suggesting that stimulation intensity primarily affects the temporal dynamics of the effect rather than the underlying mechanism. Notably, VNS did not enhance short-term learning, including online or early offline phases, even at higher intensities. Because VNS was delivered after training under dark conditions, rather than during visual stimulation or behavioral testing, the delayed enhancement is unlikely to be explained solely by acute changes in visual input, attention, arousal, or oculomotor performance. Together, these observations suggest that post-training VNS preferentially affects late-offline learning-related processes, although non-synaptic or performance-related contributions cannot be completely excluded.

Previous studies have proposed an inverted U-shaped relationship between VNS intensity and cortical plasticity, with moderate intensities producing optimal effects, and higher intensities, including those in the ∼1.0 mA range, reversing this enhancement.^42^^,^^54^ Such a relationship was not clearly observed in our study. However, we do not consider that this absence was simply attributed to the delivered intensities being too low to reach the descending limb of the inverted U-shape relationship. Previous studies reporting inverted U-shaped relationships have mainly examined cortical plasticity-related outcomes after repeated VNS-event pairing over multiple days, whereas the present study used post-training VNS delivered after each training session on day 1 and observed delayed late-offline HOKR performance enhancement.^42^^,^^54^ Moreover, nominal current amplitude alone does not define the effective biological dose of VNS, because effective fiber recruitment depends on pulse width, pulse waveform, electrode impedance, nerve-electrode coupling and implantation conditions.

Thus, the absence of a clear high-intensity reversal in our study may reflect paradigm-dependent differences in stimulation timing and stimulation history. Future studies using broader intensity ranges, direct measures of vagal afferent fiber recruitment, and plasticity-related readouts will be required to determine whether an inverted U-shaped relationship applies to VNS-induced late-offline HOKR enhancement.

The prevailing view attributes VNS-induced learning enhancement primarily to neuromodulatory systems, including cholinergic and noradrenergic pathways.^36^^,^^48^^,^^52^ In contrast, our findings highlight the contribution of vascular dynamics. Using both autofluorescence-based “shadow” imaging and plasma Alb-mScarlet labeling, we demonstrated that VNS induces robust vascular volume fluctuations in the cerebellar flocculus. Across repeated stimulation, the magnitude of these vascular oscillations was positively associated with long-term learning performance. Previous PET studies have reported VNS-induced changes in cerebral blood flow, typically interpreted as NVC.^55^^,^^56^^,^^57^ These vascular responses are generally considered secondary to neuronal activity. Another important consideration is whether the observed vascular dynamics could be explained by systemic physiological changes, such as VNS-evoked HR changes. However, the trial-dependent adaptation profile of HR changes differed from that of the vascular responses, with HR reduction becoming less pronounced during repeated trials while vascular responses persisted. This suggests that VNS-evoked vascular dynamics are unlikely to be explained solely by HR changes. Together with the possibility that neuromodulatory transmitters mediate vascular responses downstream of VNS, our findings raise the possibility that vascular dynamics may represent an additional functional dimension associated with learning, rather than merely a passive consequence of neuronal activation.

Studies of cerebellum-dependent HOKR learning have shown that repeated visual stimulation induces both behavioral learning and entrainment of vasomotion,^18^ with the magnitude of vascular oscillations correlating with learning performance. Our previous work further demonstrated that late-offline HOKR learning depends on Bergmann glia-mediated phagocytosis,^58^ a process likely associated with local metabolic demand. Together, these findings suggest that long-term memory consolidation involves structural and metabolic remodeling, and that the extent of plasticity may be modulated by the availability of surplus energy during and immediately after learning. Plasticity-related processes may be initiated within this temporal window, while their behavioral expression may emerge over subsequent days. Importantly, however, the present study does not directly establish that the late-offline HOKR performance enhancement elicited by post-training VNS is mediated primarily by memory consolidation. Additional timing-control experiments, such as pre-training or during-training VNS, together with direct manipulation of memory consolidation-related processes, will be required to test this possibility. Overall, in this framework, the likelihood of plasticity induction (i.e., metaplasticity) may be modulated by external interventions such as VNS, particularly when applied within this critical period.

One possible mechanism linking vascular dynamics to learning is neurometabolic coupling. The astrocyte-neuron lactate shuttle (ANLS) has been implicated in supporting neuronal activity during long-term memory formation.^59^^,^^60^ The cerebellum exhibits distinct metabolic features, including glycogen storage and high energetic demand in Purkinje cells,^61^ further supporting the importance of sustained metabolic support in cerebellum-dependent learning. VNS-induced vascular oscillations may contribute to this process by facilitating efficient delivery of metabolic substrates, either through astrocyte-mediated pathways such as ANLS or via direct neuronal uptake of glucose and oxygen.

Clinically, FDG-PET studies have reported region-specific changes in brain glucose metabolism following VNS in patients with epilepsy or depression,^62^^,^^63^ as well as in rodent models.^64^ However, similar studies using transcutaneous VNS (taVNS) have yielded less consistent results,^65^ suggesting differences between invasive and non-invasive stimulation or the requirement for prolonged stimulation to induce detectable metabolic changes. Thus, while VNS likely influences brain metabolism, the underlying mechanisms remain incompletely understood.

In summary, our findings suggest that, in addition to neuromodulatory mechanisms, VNS-induced vascular oscillations may represent a previously underappreciated component of brain function associated with long-term learning. These results provide a new perspective on how peripheral signals can influence central brain function through coordinated vascular and metabolic processes, and raise the possibility that endogenous peripheral signals may engage similar mechanisms linking vascular dynamics to learning.

### Limitations of the study

Several limitations should be considered. First, although vascular oscillations were strongly associated with learning performance, causality was not directly tested. Second, metabolic dynamics were not directly measured in this study. Future experiments using genetically encoded metabolic sensors combined with fiber photometry will be required to directly assess changes in ATP, lactate, and pyruvate.^9^^,^^66^ In addition, direct measurements of arterial oxygen saturation, arterial PO_2_, and brain tissue PO_2_ will be important to clarify the contribution of oxygenation changes to VNS-evoked vascular dynamics. Vascular optogenetic approaches may enable causal manipulation of vascular dynamics to determine their role in learning.^67^ The contribution of astrocytes and ANLS could also be directly examined through pharmacological manipulation of monocarboxylate and glucose transporters.

## Resource availability

### Lead contact

Requests for further information and resources should be directed to and will be fulfilled by the lead contact, Ko Matsui (matsui@tohoku.ac.jp).

### Materials availability

This study did not generate new unique reagents.

### Data and code availability

All the study data are included in the main text and supplemental information. Any additional information required to reanalyze the data reported in this study is available from the lead contact upon request. Further information and requests for resources, reagents, and codes (Python, MATLAB, and R) should be directed to and will be fulfilled by the lead contact.

## Acknowledgments

We are thankful to all members of the K.M. laboratory for their invaluable assistance at every stage of the experiments. This work was supported by Neuro Global Program in Tohoku University (to J.U.C.), Grant-in-Aid for Early-Career Scientists 22K15218, 24K18234 (to Y.I.), Grant-in-Aid for Scientific Research (C): 26K09537 (to Y.I.), Grant-in-Aid for Transformative Research Areas (A) “Glial Decoding”: 20H05896 (to K.M.), “Biology of Behavior Change”: 23H04659, 25H01713 (to K.M.), “pH-Responsive Biology”: 26H01665 (to K.M.), Grant-in-Aid for Scientific Research on Innovative Areas “Brain Information Dynamics”: 18H05110, 20H05046 (to K.M.), Grant-in-Aid for Scientific Research (B): 19H03338, 22H02713, 25K02373 (to K.M.), 10.13039/100007449Takeda Science Foundation (to K.M.), the 10.13039/100008732Uehara Memorial Foundation (to K.M.), and 10.13039/501100005927Daiichi Sankyo Foundation of Life Science (to K.M.).

## Author contributions

Conceptualization, J.U.C., Y.I., and K.M.; methodology, J.U.C., Y.I., and K.M.; investigation, J.U.C.; formal analysis, J.U.C. and K.M.; data curation, J.U.C.; writing – original draft, J.U.C. and K.M.; writing – review and editing, Y.I.; supervision, Y.I. and K.M.; funding acquisition, Y.I. and K.M.

## Declaration of interests

The authors declare no competing interests.

## STAR★Methods

### Key resources table

**Table:** 

REAGENT or RESOURCE	SOURCE	IDENTIFIER
**Bacterial and virus strains**		

AAV8- P3-Albumin-mScarlet	Wang et al.^45^	Addgene ID: 183461

**Chemicals, peptides, and recombinant proteins**		

Sodium nitroprusside (SNP)	NACALAI TESQUE	Cat # 31620–62

**Experimental Models: Organisms/Strains**		

Mouse: C57BL/6 J	CLEA Japan	RRID:SCR_026053

**Software and algorithms**		

AxoGraph	AxoGraph Scientific	RRID:SCR_014284
Spike2 7.20	Cambridge Electronic Design	RRID:SCR_000903
Fiji (ImageJ)	NIH	RRID:SCR_002285
MATLAB 2021b	Mathworks	RRID:SCR_001622
Python 3.8	Python Software Foundation	RRID:SCR_008394
R	R Foundation for Statistical Computing	RRID:SCR_001905
GraphPad Prism 10.0	GraphPad Software	RRID:SCR_002798

### Experimental model and study participant details

All animal experiments were conducted in accordance with the Regulations for Animal Experiments and Related Activities at Tohoku University and were approved by the Institutional Animal Care and Use Committee of Tohoku University (2019LsA-017). All efforts were made to minimize animal suffering and to reduce the number of animals used.

All mice used in this study were male C57BL/6 J mice aged 8–13 weeks. Animals were housed under controlled conditions (23°C–26°C, 40–60% humidity) on a 12-h light/dark cycle, with food and water available *ad libitum*. After weaning, mice were group-housed with same-sex littermates (no more than six per cage) to promote environmental enrichment. Following surgical procedures, mice were housed individually. Behavioral experiments were conducted during the light phase.

### Method details

#### Surgical procedures

All surgical procedures were performed under anesthesia using a mixture of medetomidine hydrochloride (0.75 mg/kg; Domitor; Nippon Zenyaku Kogyo Co., Ltd., Japan), midazolam (4 mg/kg; Sandoz Inc., Japan), and butorphanol tartrate (5 mg/kg; Vetorphale; Meiji Seika Pharma Co., Ltd., Japan). Body temperature was maintained at ∼36.5°C using a homeothermic plate system (GEX Corporation Co., Ltd., Japan). Mice were allowed to recover for at least 10 days before experiments.

##### Vagus nerve cuff electrode design

A customized cuff electrode for vagus nerve stimulation (VNS) was developed in our laboratory based on a previously established design. The detailed configuration of the cuff electrode is illustrated in Figures 1A and 1B.

The electrode consisted of a biocompatible cylindrical silicone tube (Unique Medical Co., Ltd., Japan) coupled with two stainless-steel wires (AS 631, Cooner Wire Company, USA). The exposed anterior segments of the wires served as electrode contact sites along the inner surface of the silicone tube. The cuff dimensions were as follows: inner diameter, 0.5 mm; outer diameter, 1.5 mm; cathode–anode spacing, 1.5 mm; and total length, 2.0 mm. The proximal ends of the stainless-steel wires were connected to a 4-pin connector, which interfaced with the external stimulator.

##### Cuff electrode implantation

Based on anatomical studies indicating that the left vagus nerve contains a higher proportion of afferent fibers (approximately 80%) compared to efferent fibers,^27^ the cuff electrode was implanted on the left cervical vagus nerve to preferentially engage afferent pathways projecting to the central brain.

Under deep anesthesia, the neck-to-chest region was shaved and disinfected with repeated alcohol swabs. A 1.5–2 cm midline incision was made from the manubrium to the jawline using blunt dissection. The left submandibular gland (SMG) was gently separated from the surrounding connective tissue and retracted laterally. The connective tissue between the left sternocleidomastoid and sternothyroid/omohyoid muscles was carefully dissected to expose the carotid sheath. A small pair of surgical retractors was used to maintain muscle separation. The left cervical vagus nerve was identified, and a 4–5 mm segment was carefully isolated. A customized cuff electrode was positioned around the nerve, ensuring circumferential contact of both electrode sites. The cuff electrode was then secured to the sternocleidomastoid muscle using a silk suture (Figure 1B).

##### Implantation of electrocardiogram electrodes

To monitor VNS-induced changes in heart rate, a pair of electrocardiogram (ECG) electrodes constructed from stainless-steel wires (total length, 5.0 mm; AS 633, Cooner Wire Company, USA) was implanted subcutaneously and sutured onto the chest muscles (Figure 1A, black spots). The proximal ends of the wires were configured similarly to those of the cuff electrode and connected to the same 4-pin connector, which was interfaced with an amplifier for signal acquisition.

##### Optical fiber implantation

To monitor vascular dynamics in the vicinity of the cerebellar flocculus *in vivo*, a fiber photometry approach was employed. The cerebellar flocculus is known to play a key role in horizontal optokinetic response (HOKR) motor learning.^30^

A glass optical fiber (core diameter, 400 μm; numerical aperture, 0.39; cat. no. FT400UMT; Thorlabs) equipped with a ceramic ferrule (external diameter, 2.5 mm; Kyocera Co., Japan) was used. The optical fiber was positioned above the cerebellar flocculus at coordinates 5.85 mm posterior and 3.0 mm lateral to bregma, and implanted to a depth of 1.50 mm from the cortical surface (Figure 3B). The implanted optical fiber was secured using dental resin (Super Bond C&B; Sun Medical Co., Ltd., Japan) mixed with charcoal particles to prevent light penetration.

After completion of the experiments, mice were transcardially perfused with 4% paraformaldehyde in PBS. Brains were sectioned into 100 μm-thick coronal slices using a vibratome (VT1000S; Leica Microsystems, Germany), and fiber placement was confirmed histologically.

##### Head-fixation system implantation

For pupil imaging and horizontal optokinetic response (HOKR) measurements, a head-fixation system was used to maintain the mouse’s head in a stable position facing the visual stimulus display. During experiments, the body of the mouse was gently restrained in an upward-slanted plastic cylinder.

For implantation, anesthesia was induced and the mouse was positioned in a stereotaxic apparatus. Stereotaxic coordinates were determined based on a mouse brain atlas.^68^ The surgical area was shaved and disinfected with repeated alcohol swabs. A customized screw (6 mm head diameter, 10 mm length; Matsumoto Industrial Co., Ltd., Japan) was attached to the skull between bregma and lambda using dental resin (Super Bond C&B; Sun Medical Co., Ltd., Japan), forming a stable head-fixation mount.

##### Fluorescent positive labeling of blood volume

To directly assess changes in vascular volume using *in vivo* fiber photometry, blood vessels were fluorescently labeled by expressing an albumin–mScarlet (Alb-mScarlet) fusion protein in hepatocytes via adeno-associated virus serotype 8 (AAV8).^45^ Details of the AAV8 vector carrying the P3-Alb-mScarlet construct have been described previously.^46^ Briefly, the purified AAV8 vector was diluted in phosphate-buffered saline (PBS) to a final concentration of 2 × 10^12^ vg/mL, and 300 μL of the solution was administered intraperitoneally (i.p.) to each mouse after cuff electrode implantation surgery.

Vascular imaging experiments were performed after a 3-week expression period to ensure stable fluorescence signals in the circulation (Figure 4A). The Alb-mScarlet fusion protein was secreted into the bloodstream and circulated systemically, thereby enabling positive labeling of blood vessels.

##### Surgical care

Following surgical procedures, including implantation of cuff electrodes for cervical vagus nerve stimulation (VNS), atipamezole hydrochloride (0.75 mg/kg; Antisedan; Nippon Zenyaku Kogyo Co., Ltd., Japan) was administered to reverse the effects of medetomidine used during anesthesia. Butorphanol administered prior to surgery served as an analgesic, providing postoperative pain relief for several hours.

After surgery, mice were housed individually in home cages placed on a heating plate (HP-4530 N; AS ONE Corporation, Japan) maintained at ∼32°C during the recovery period (at least 10 days). Gel-form water and nesting material were provided to ensure hydration and environmental enrichment. Animals were monitored twice daily for signs of distress or health deterioration. If any signs of distress or significant weight loss were observed, animals were humanely euthanized. Only animals that passed this postoperative screening were included in subsequent experiments.

At the conclusion of the experiments, animals were either euthanized by inhalation of vaporized isoflurane (∼1 mL per 1 L of chamber volume), inducing an overdose of anesthesia, or transcardially perfused with 4% paraformaldehyde in PBS (Lot No. L5K0764; NACALAI TESQUE Inc., Japan) for subsequent histological analysis. Death was confirmed by cessation of respiration.

#### Vagus nerve stimulation protocol

Vagus nerve stimulation (VNS) was delivered using a constant-current stimulator (DS3; Digitimer, UK) controlled by a pulse generator (Master-8; A.M.P.I., Israel). Stimulation consisted of 250 μs pulses delivered at 30 Hz with amplitudes of 0.6 or 1.5 mA for 10 s per train. VNS trains were repeated at 40-s intervals (start-to-start) for a total of 30 repetitions (Figure 1C). These stimulation parameters were selected based on previous studies demonstrating reliable activation of vagal afferent pathways.

In both control and VNS groups of the HOKR experiments, VNS was delivered at the end of each experiment to confirm pupil dilation and heart rate responses as physiological indicators of effective VNS delivery.

All stimulation pulses generated by the Master-8 pulse generator were recorded using Spike2 7.20 software (Cambridge Electronic Design, UK) at a sampling frequency of 2 kHz and digitized via an A/D converter (CED Micro1401-3 with ADC12 top box, Cambridge Electronic Design, UK). Because the native pulse width was too short to be reliably detected, additional reference pulses (10 ms width) were generated in parallel by the Master-8 and recorded simultaneously to provide a precise temporal reference for VNS delivery.

In a separate set of experiments, a graded stimulation protocol was applied to assess pupil dilation responses. In each VNS train, stimulation frequency, duration, and pulse width were identical to those used in the HOKR experiments. Stimulation amplitudes were varied across four levels: 0.2 mA (low), 0.6 and 0.8 mA (intermediate), and 1.5 mA (high). At each amplitude, stimulation polarity was alternated by adjusting the orientation of the cathode in the constant-current stimulator (DS3; Digitimer, UK), with the cathode positioned either cephalad or caudad (Figure S4A).

#### Acquisition and assessment of ECG and pupil responses

To validate effective VNS delivery via the implanted cuff electrode, ECG and pupil responses were recorded. For ECG recordings, signals were amplified using a DAM50 extracellular amplifier (World Precision Instruments, USA) and band-pass filtered between 10 Hz and 1 kHz. The signals were then digitized using an A/D converter (CED Micro1401-3 with ADC12 top box, Cambridge Electronic Design, UK) and recorded with Spike2 7.20 software (CED) at a sampling frequency of 5 kHz. For pupil measurements, the right eye of the mouse was illuminated by two infrared (IR) LEDs. An angled hot mirror, which selectively reflects IR light, was positioned between the eye and the monitor (Figure 2A). The reflected IR image of the eye was captured using a CCD camera (DN3V-30BU; Shodensha Inc., Japan) at 30 fps with the IR-cut filter removed.

##### Eye movement tracking

The reflected infrared (IR) image of the right eye, acquired using the camera described in the main manuscript, was analyzed using DeepLabCut^69^ to track five key points: the pupil center and four peripheral edge points (Figure 2B).

For network training, 13 videos obtained from five mice were used. A total of 280 frames were selected using a k-means clustering algorithm to construct the training dataset. The network was trained for 650,000 iterations per training session, and this training procedure was repeated three times. The trained network was then applied to extract pupil positions across all experimental sessions.

To ensure data quality, coordinates with likelihood values below 0.8 were excluded and treated as missing values. Frames in which the pupil center and all edge points were simultaneously undetected (e.g., due to blinks or partial occlusion) were classified as invalid and excluded from further analysis.

##### Quantification of VNS-evoked pupil response

Pupil size was estimated using a circular approximation to ensure robustness under partial point detection and to minimize data loss. The radius was defined as the average distance between the pupil center and available edge points. When both horizontal (left–right) or vertical (top–bottom) pairs were available, their averaged distances were used; otherwise, single-point distances were used when the likelihood of the center coordinate exceeded the threshold. Outliers were identified using an interquartile range (IQR)-based method, whereby values falling outside 1.5 × IQR from the first and third quartiles were classified as outliers and replaced with interpolated estimates. To reduce high-frequency noise while preserving signal dynamics, the cleaned pupil area time series was smoothed using a Savitzky–Golay filter (window length = 7 frames, polynomial order = 3).

VNS-evoked pupil responses were quantified using AxoGraph (AxoGraph Scientific, Australia). During each VNS train, pupil size was normalized to baseline, defined as the mean pupil size during the 2.0 s preceding VNS onset (set to 1.0). The peak pupil size was identified within a 10 s window following VNS onset.

To ensure accurate temporal alignment between stimulation and pupil dynamics, two additional IR LEDs were mounted below the IR camera as timing markers. These LEDs flickered synchronously with VNS onset and were reflected on the cornea, with the flicker spots positioned on both sides of the pupil to avoid interference with tracking (flicker duration: 100 ms). Based on these markers, latency to peak dilation was determined for each VNS train.

For validation of effective VNS in behavioral experiments, animals were excluded if the relative pupil size exceeded 1.5 fewer than 25 times across 30 VNS trains (0.6 mA and 1.5 mA conditions), indicating insufficient VNS efficacy. In addition, after the probe test on Day 5, mice from all groups (including the sham group) received five VNS trains at 1.5 mA; animals were excluded if the response exceeded 1.5 fewer than three times, indicating loss of cuff electrode efficacy.

For graded assessment of VNS-evoked pupil responses, each 10 s VNS train was delivered three times per amplitude and stimulation polarity within a single series (24 trains; interval: 30 s). This series was repeated three times at intervals of at least 1 h, resulting in at least nine repetitions per condition. Peak pupil responses were averaged to obtain a single value per condition for each mouse.

##### Preprocessing of ECG data

To remove stimulation-induced artifacts from the ECG trace, a custom Python-based time-shifted temporal averaging method was employed (Figure S2). The stimulation timing trace, recorded synchronously with the ECG signal, was used to identify the onset of the first stimulation pulse, which was defined as time zero (0 s). Based on the stimulation frequency (30 Hz), multiple temporally shifted versions of the ECG signal were generated by applying small offsets (−66, −33, +33, and +66 ms) to the time axis while preserving the signal values. This procedure maintained stimulation-locked artifacts while disrupting the temporal alignment of cardiac pulse components (QRS complexes).

All shifted and original signals were aligned onto a common time axis using nearest-neighbor mapping. At each time point, values across all signals were averaged to estimate the stimulation-induced artifact. Because the artifact was temporally locked to stimulation, it was preserved during averaging, whereas cardiac components, which varied in phase across shifts, were attenuated. The estimated artifact was then subtracted from the original ECG trace to isolate the underlying cardiac activity. Finally, the time axis was adjusted such that the earliest time point was set to zero for subsequent analysis (Figure S2B).

##### BPM calculation procedure

The artifact-subtracted ECG signal was processed using a custom Python implementation based on the Pan–Tompkins algorithm to extract heart rate.^70^ The ECG signal was first band-limited using a two-step filtering procedure. A notch filter centered at 50 Hz was applied to remove power line interference, followed by a band-pass Butterworth filter (10–120 Hz) to isolate QRS complex components. Both filters were applied using zero-phase forward–backward filtering to avoid phase distortion. The filtered ECG signal was then squared and subjected to moving window integration (MWI) using a 20 ms sliding window. To improve robustness against extreme peaks, the MWI signal was clipped at the 99.5th percentile prior to peak detection. R-peaks were detected using an adaptive thresholding approach with constraints on peak amplitude and minimum inter-peak interval.

RR intervals were calculated as the temporal differences between successive R-peaks. To correct for artifacts and ectopic beats, the RR interval series was further processed using a Kubios-style automatic correction procedure, including physiological range filtering, sudden change detection, local median-based outlier detection, ectopic beat identification, and interpolation-based reconstruction.^71^ Finally, heart rate (BPM) was calculated as the inverse of the RR interval (BPM = 60/RR; Figure S2C).

##### Analysis of VNS-induced heart rate reduction

VNS-induced heart rate (HR) reduction was quantified using AxoGraph (AxoGraph Scientific, Australia). For each VNS train, HR was normalized to baseline, defined as the mean HR during the 5.0 s preceding VNS onset (set to 1.0). The HR response during VNS was quantified as the mean normalized HR over a 10 s window following VNS onset, excluding the initial 0.5 s to avoid transient fluctuations. The post-VNS response was defined as the mean normalized HR within 5 s after the end of the VNS train.

#### Fiber photometry

Fiber photometry was used to enable simultaneous monitoring of vascular-related fluorescence signals *in vivo*. A glass optical fiber equipped with a ceramic ferrule was implanted above the cerebellar flocculus and connected via a ferrule-to-ferrule interface using bronze split sleeves (ADAL4-5; Thorlabs, USA).

A custom-made fiber photometry system (Lucir, Japan) was used. Autofluorescence alone or combined with Alb-mScarlet signals from blood vessels^45^ were recorded using dedicated optical configurations. The optical setups and filter sets used for autofluorescence (Figure 3C) and Alb-mScarlet (Figure 4B) measurements are described below. Excitation light was delivered from an SPECTRA X Light Engine (Lumencor, USA), and emitted fluorescence was collected through the same optical fiber and detected using photomultiplier tubes (PMTs; PMTH-S1-CR316-02; Zolix Instruments Co., Ltd., China) equipped with current-to-voltage amplifiers (HVC1800; Zolix Instruments Co., Ltd., China). Signals were digitized using an A/D converter (CED Micro1401-3 with ADC12 top box, Cambridge Electronic Design, UK) and recorded with Spike2 7.20 software (CED) at a sampling rate of 2 kHz. Excitation light was delivered in 20 ms pulses at 100 ms intervals, and signals were averaged within each pulse (effective sampling rate: 10 Hz).

##### Optics

For selective excitation of autofluorescence, 440 nm light filtered through a band-pass filter (ET436/20×; Chroma, USA) was used. A dichroic mirror (T495lpxr; Chroma, USA) directed the excitation light toward the mouse and transmitted emitted autofluorescence. Emission light was further filtered using a band-pass filter (FF01-550/88; Semrock, USA) (Figure 3C).

For simultaneous excitation of Alb-mScarlet and autofluorescence, light at 575 nm and 440 nm, filtered through a multi-bandpass filter (69008×; Chroma, USA), was used. A multi-band beamsplitter (69008bs; Chroma, USA) directed excitation light toward the mouse and transmitted emitted fluorescence. Emission signals from Alb-mScarlet and autofluorescence were separated using a dichroic mirror (T590lpxr; Chroma, USA). The separated emission light was further filtered using band-pass filters (ET645/75m; Chroma, USA) for Alb-mScarlet and (ET537/29m; Chroma, USA) for autofluorescence (Figure 4B).

##### Fluorescence signal data processing and analysis

All fluorescence signals were analyzed offline using AxoGraph (AxoGraph Scientific, Australia) and custom scripts written in R. Signals were converted into intensity traces and visualized using AxoGraph. Unless otherwise stated, fluorescence traces were low-pass filtered at 1 Hz for visualization.

Peak amplitudes and their corresponding time points were identified within a window from 5 s before to 25 s after VNS onset (defined as 0 s). Absolute area under the curve (AUC) values and trial-by-trial autofluorescence response heatmaps were computed using custom R code. The temporal relationship between Alb-mScarlet and autofluorescence signals was evaluated using cross-correlation analysis. The lag time corresponding to the negative peak around 0 s in the cross-correlation function was defined as Δt.

To quantify the magnitude of divergent vascular oscillations, autofluorescence traces were band-pass filtered at 0.01–0.5 Hz, squared, and integrated. The slope within each period and the cumulative autofluorescence integral during the late post-training period (1200–1210 s) were calculated. Finally, the slopes calculated for each period across the four training sessions were averaged to obtain the mean slope for each period. Meanwhile, the cumulative autofluorescence integrals during the late post-training period from the four training sessions were summed to obtain the total cumulative autofluorescence integral.

#### Pharmacological dilation of blood vessels

Sodium nitroprusside (SNP) was used to induce vasodilation as a pharmacological manipulation of vascular dynamics.^72^ SNP (NACALAI TESQUE Inc., Japan) was initially dissolved in saline at a concentration of 150 mg/mL and aliquoted into 3 μL portions. Immediately before administration, a 3 μL aliquot (450 μg SNP) was diluted with saline to a final volume of 600 μL (0.75 mg/mL). From this solution, 300 μL (225 μg SNP) was injected subcutaneously into the cervical region.

#### HOKR experiments

For horizontal optokinetic response (HOKR) experiments, sinusoidal oscillations of vertical stripes were presented on two PC monitors arranged orthogonally around the mouse (Figure 2A). Visual stimuli were generated and controlled using a custom MATLAB program (MathWorks, USA), as described previously.^31^ Stimulus parameters were set to an amplitude of 17° (peak-to-peak), a temporal frequency of 0.25 Hz, and a spatial frequency of 6.4°/cycle.

Eye movements were recorded using an infrared imaging system and analyzed with DeepLabCut^69^ to track pupil position. Eye movement angles and amplitudes were derived from the tracked pupil coordinates as described below.

##### Eye movement calculation

Eye position was converted to eye direction angle relative to the camera using a method previously described by Sakatani and Isa.^73^ To accurately quantify eye movement amplitude, raw eye movement traces were preprocessed using custom Python and MATLAB scripts.

Saccadic eye movements were identified as events in which the change in eye position between consecutive frames (1/30 s) exceeded 1° of visual angle. These saccadic events were excluded from further analysis. Slow drift components were removed using a band-pass filter (0.1–0.5 Hz) (Figure 2C). Eye movement amplitude was calculated as the difference between the mean of peak maxima and the mean of peak minima.

##### Validity criteria in HOKR experiments

HOKR relative gain was calculated by normalizing eye movement amplitude to that measured at the beginning of the first training session. Initial learning performance was defined relative to the visual stimulus.

Data from four mice were excluded because they did not reach a predefined criterion of 0.2 in initial HOKR performance. To validate effective VNS delivery during HOKR experiments, mice in Groups B and C were excluded if no pupil dilation was observed during post-training VNS following the first training session. In such cases, subsequent training sessions were discontinued (Group B: four mice; Group C: six mice). Similarly, in Group A, mice were excluded if no pupil dilation was detected after the probe test on Day 5 (three mice). Fisher–Freeman–Halton exact tests revealed no significant group-dependent differences in postoperative mortality (*p* = *0*.*942*), exclusion based on the initial HOKR criterion (*p* = *1*.*000*), or exclusion due to absent pupil dilation (*p* = *0*.*545*). These analyses did not reveal evidence of group-dependent attrition or exclusion, although they do not formally establish MCAR or exclude MNAR. The full contingency tables and exact-test results are provided in the Data S1.

### Quantification and statistical analysis

All data are presented as mean ± standard error of the mean (SEM). Statistical analyses were performed using GraphPad Prism (version 10.0; GraphPad Software, USA).

Normality was assessed using the Shapiro–Wilk test. For normally distributed data, a one-sample *t* test was used to compare the sample mean against a hypothesized value (set to 1.0 or 0.0 in this study). Simple linear regression was used to evaluate correlations between variables, with correlation coefficients (r) and *p*-values reported. Comparisons between two groups were performed using a two-sided unpaired *t* test. Welch’s correction was applied when the assumption of equal variance was violated. Paired comparisons were conducted using a two-sided paired Student’s *t* test when appropriate. Comparisons among more than two groups were performed using one-way ANOVA followed by Tukey’s post hoc test. Two-way repeated-measures ANOVA followed by Tukey’s or Bonferroni’s multiple-comparisons tests was used to assess the effects of two factors when appropriate. For repeated-measures datasets with unequal group sizes, including the HOKR learning data, a mixed-effects model fitted by restricted maximum likelihood (REML) was used. Training session, VNS condition, and their interaction were included as fixed effects, which were assessed using Type III tests. Post hoc comparisons were performed using Tukey’s multiple-comparisons test.

Statistical significance was set at *p* < 0.05. Detailed information on statistical tests, sample sizes, and *p*-values is provided in the figure legends. Complete statistical results are summarized in the Data S1.

## Supplemental information

Document S1. Figures S1–S9

Data S1. Supplemental statistical tables
