# Supplementary material for: Vagal nerve stimulation induces vascular oscillations and enhances long-term learning
Source: iScience. 2026 Aug 25;29(9):117413. doi: 10.1016/j.isci.2026.117413 (PMC13594465; doi:10.1016/j.isci.2026.117413)
Supplement: Supplementary file 1 [file mmc1.pdf]

iScience, Volume *29*

Supplemental information

Vagal nerve stimulation induces vascular oscillations and enhances long-term learning

Junyu U. Chen (陳俊宇), Yoko Ikoma (生駒葉子), and Ko Matsui (松井広)

Supplementary Information

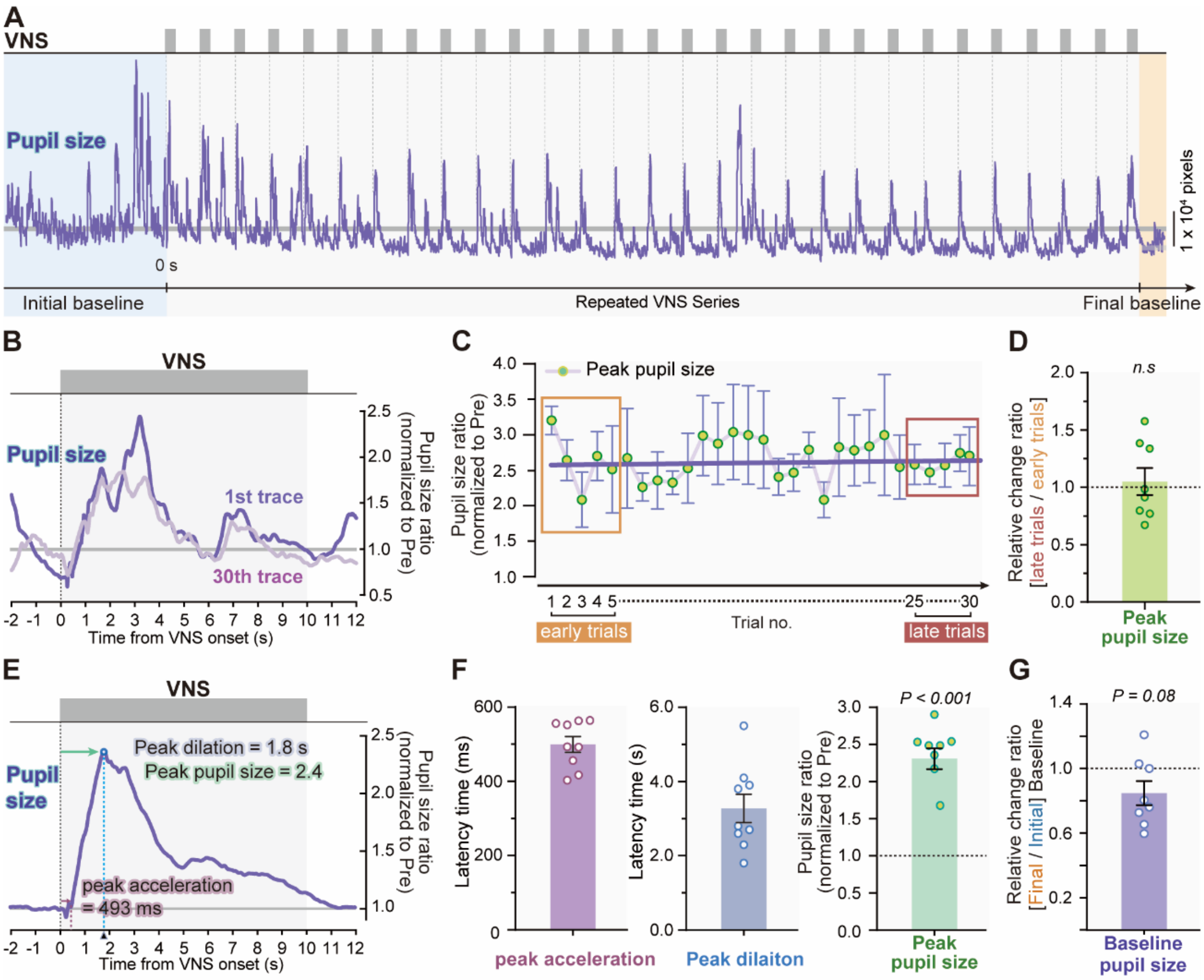

Figure S1: VNS reliably evokes pupil dilation across repeated stimulation trials. **A,** Representative pupil size trace during a VNS series. **B,** Pupil dilation traces from the first and thirtieth VNS trials. **C,** Peak pupil size ratios across VNS trials. Trials 1–5 and 25–30 were defined as early and late trials, respectively. **D,** Relative change in peak pupil size ratio between early and late trials (one-sample t-test, $t_{(7)} = 0.4325$, $p = 0.68$, n.s.; n = 8, mice from the 1.5 mA VNS group). **E,** Average pupil dilation traces across the VNS series, showing a delayed onset following VNS initiation, rapid peak, and return toward baseline. **F,** Quantification of pupil dilation characteristics, including peak pupil size and latency to peak acceleration (peak pupil size: $t_{(8)} = 9.366$, $p < 0.001$; n = 9, mice from the 1.5 mA VNS group). **G,** Baseline pupil size remained stable across repeated VNS trials ($t_{(7)} = 2.039$, $p = 0.08$, n.s.; n = 8), indicating no significant drift over time. Data are presented as mean ± SEM.

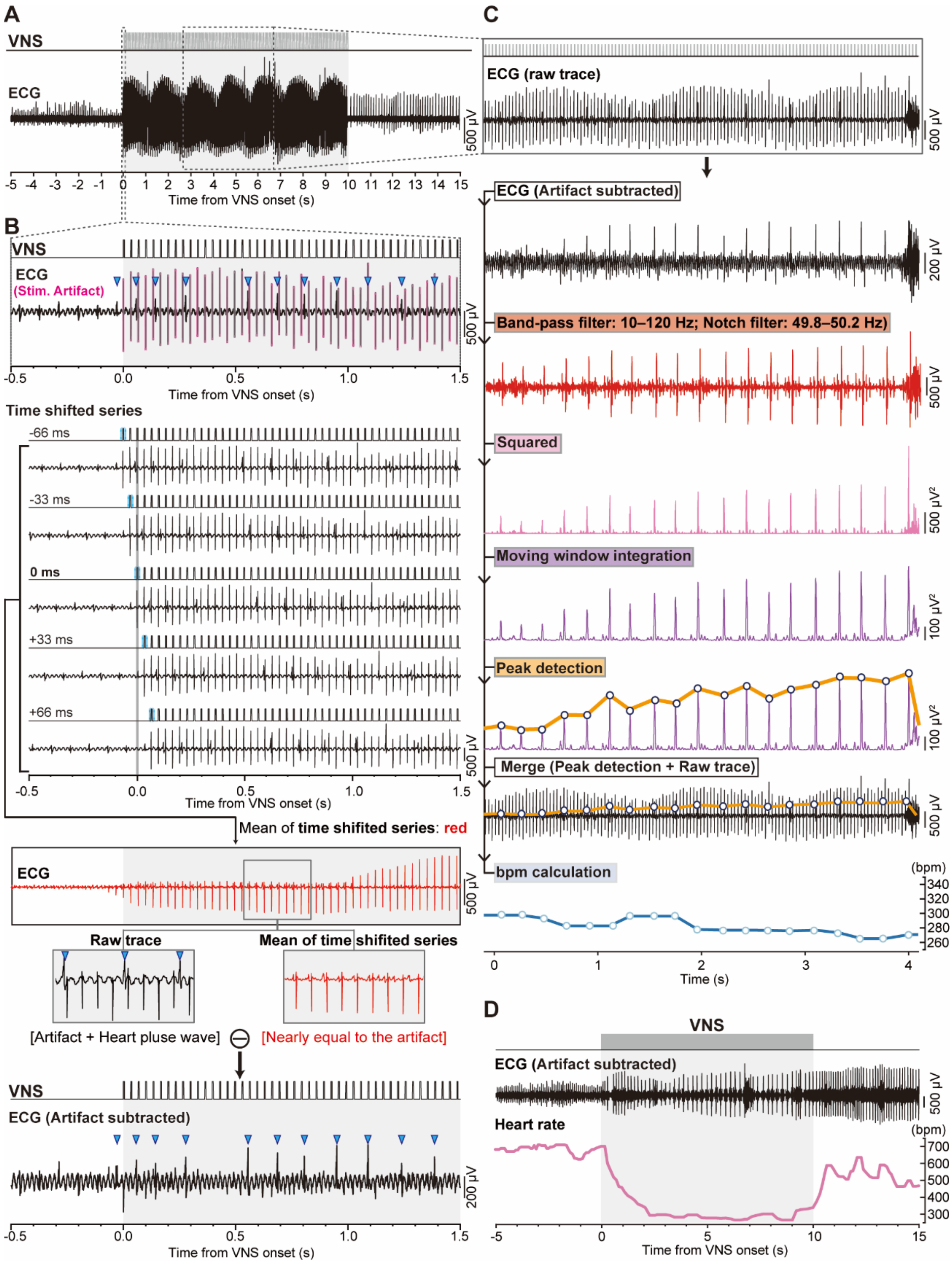

Figure S2: Removal of stimulation artifacts and heart rate detection using a Pan–Tompkins–based algorithm. A, Representative ECG trace showing large stimulation artifacts during VNS delivery. **B,** Enlarged view at the onset of VNS. Stimulation artifacts are highlighted

in red, and cardiac pulse waves are indicated by blue arrows. To isolate stimulation artifacts, the ECG trace was aligned to the first VNS pulse (time 0 s) and temporally shifted (−66, −33, 0, +33, and +66 ms), generating five time-shifted traces (middle). Averaging these traces produced a mean time-shifted trace that predominantly represented stimulation artifacts. Subtraction of this mean trace from the raw ECG signal yielded an artifact-subtracted ECG trace (bottom), in which cardiac pulse waves were clearly resolved. **C,** Enlarged ECG segment during VNS (top) and corresponding artifact-subtracted trace. The cleaned ECG signals were processed using a Pan–Tompkins–based algorithm, including band-pass filtering (10–120 Hz), notch filtering (49.8–50.2 Hz), signal squaring, and moving-window integration. R peaks were detected, and inter-peak intervals were used to calculate heart rate (bpm). **D,** Representative artifact-subtracted ECG trace and corresponding heart rate trace, showing a transient reduction in heart rate during VNS.

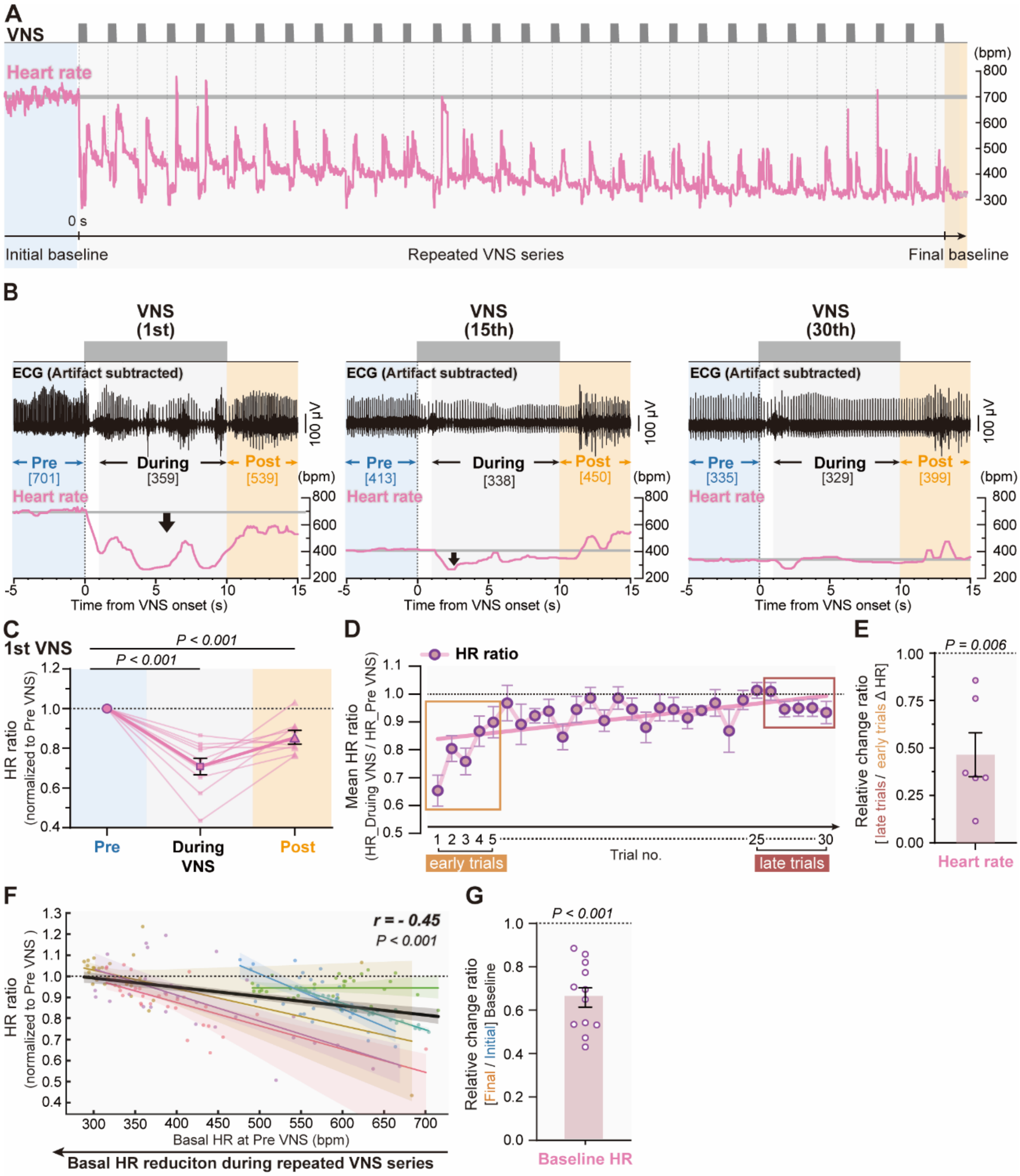

Figure S3: VNS-evoked heart rate changes across repeated stimulation trials. A, Representative heart rate trace during a VNS series. **B,** Heart rate traces from the first, fifteenth, and thirtieth VNS trials. **C,** Heart rate changes during the first VNS trial across animals. One-way repeated-measures ANOVA showed a significant effect ($F_{(1.51,\ 13.62)}$ = 28.59, p < 0.001), followed by Dunnett's multiple comparisons test (Pre vs. During VNS: 1.000 vs. 0.7085 ± 0.0410, p < 0.001; Pre vs. Post VNS: 1.000 vs. 0.8537 ± 0.0261, p < 0.001; mean ± SEM; n = 10, 1.5 mA VNS group). **D,** Heart rate ratios during each VNS trial across the stimulation series. Definitions of early and

late trials are shown in Fig. S1**C**. **E,** Relative change in heart rate ratio between early and late trials (one-sample t-test, $t_{(5)} = 4.625$, $p = 0.006$; n = 6). **F,** Relationship between baseline heart rate and heart rate ratio during VNS. A significant negative correlation was observed ($r = -0.45$, $p < 0.001$, linear regression; n = 6). Points of the same color represent data from the same animal. **G,** Relative change in baseline heart rate across trials (one-sample t-test, $t_{(11)} = 7.620$, $p < 0.001$; n = 12, including mice from 0.6 mA and 1.5 mA VNS groups). Data are presented as mean ± SEM.

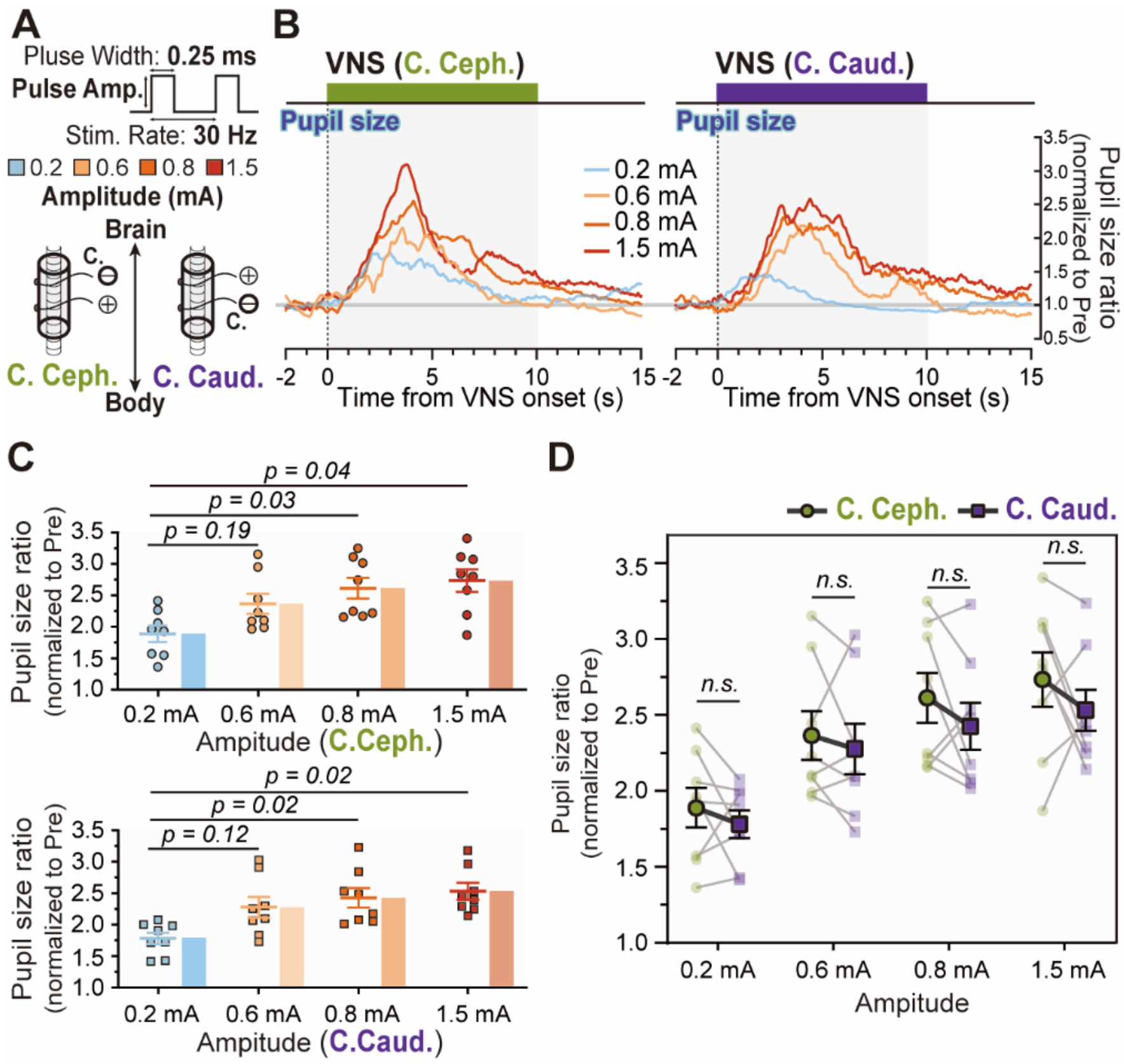

Figure S4: VNS-evoked pupil dilation depends on a two-dimensional stimulation parameter space. **A,** Schematic illustration of VNS stimulation parameters. The two-dimensional parameter space consisted of graded stimulation amplitudes (0.2–1.5 mA) and stimulation polarity, defined by cathode placement either cephalad (forest green) or caudad (deep purple). **B,** Representative pupil dilation traces evoked by different combinations of stimulation amplitude and polarity. **C,** Peak pupil dilation ratios across stimulation amplitudes. One-way repeated-measures ANOVA followed by Tukey's multiple comparisons test (see Supplementary Statistical Tables). **D,** Peak pupil dilation ratios across stimulation amplitudes and polarities. Two-way repeated-measures ANOVA followed by Bonferroni's multiple comparisons test (see Supplementary Statistical Tables). Data are presented as mean ± SEM (n = 8).

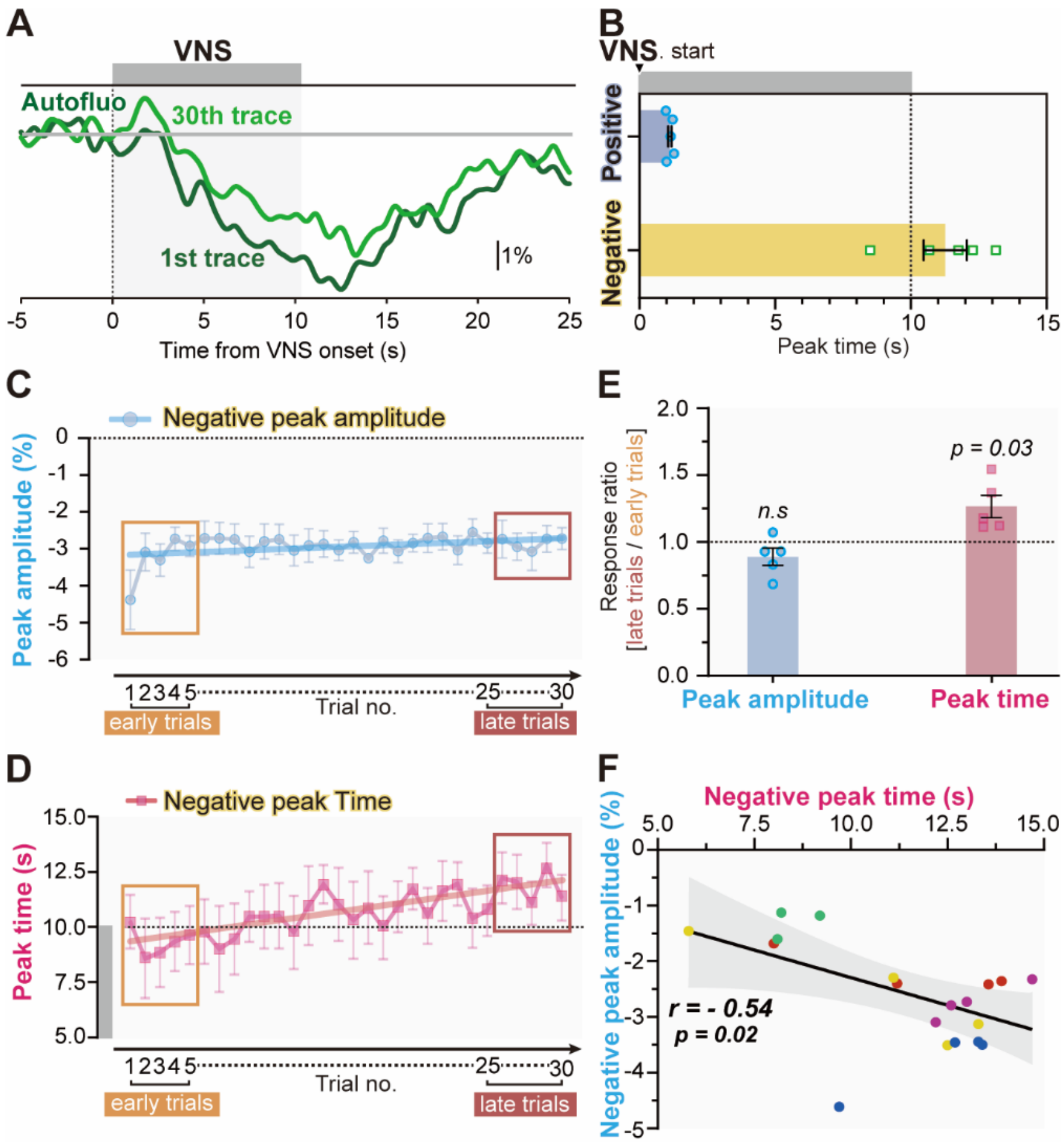

Figure S5: VNS-evoked transient autofluorescence fluctuations across repeated stimulation trials. A, Autofluorescence traces from the first and thirtieth VNS trials. **B,** Average onset times of positive and negative peaks of the autofluorescence signal. **C-F,** Because the dominant effect of VNS-evoked autofluorescence fluctuations was observed during the negative phase, subsequent analyses focused on the negative phase. **C,** Negative peak amplitudes across VNS trials. **D,** Negative peak times across VNS trials. **E,** Relative changes in negative peak amplitude and peak time between early and late trials (one-sample t-tests; peak amplitude: $t_{(4)} = 1.733$, $p = 0.16$, n.s.; peak time: $t_{(4)} = 3.176$, $p = 0.03$; n = 5). Definitions of early and late trials are shown in Fig. S1**C**. **F,** Relationship between negative peak amplitude and peak time. A significant negative correlation was observed (r = −0.54, $p = 0.02$, linear regression). Each data point represents the average of 30 trials from a single VNS series (n = 19 episodes from 5 mice). Colors indicate individual animals. Data are presented as mean ± SEM.

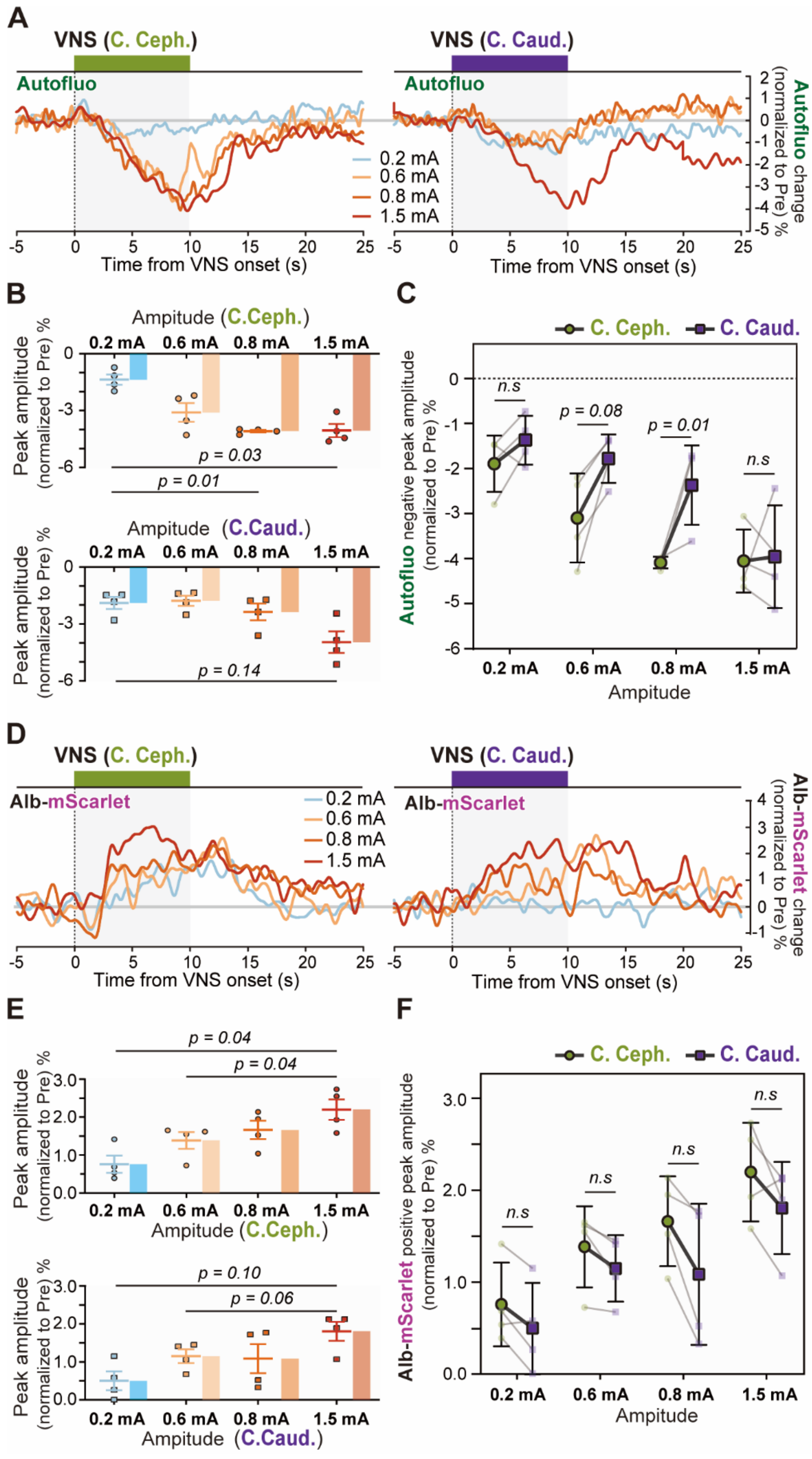

Figure S6: Autofluorescence and Alb–mScarlet responses across a two-dimensional VNS stimulation parameter space. A, Representative autofluorescence traces under different

stimulation conditions. **B,** Negative peak amplitudes of autofluorescence across stimulation amplitudes within each polarity condition (one-way repeated-measures ANOVA followed by Tukey's multiple comparisons test; see Supplementary Statistical Tables). **C,** Negative peak amplitudes of autofluorescence across stimulation amplitudes and polarities (two-way repeated-measures ANOVA followed by Bonferroni's multiple comparisons test; see Supplementary Statistical Tables). **D,** Representative Alb–mScarlet traces under different stimulation conditions. **E,** Peak amplitudes of Alb–mScarlet across stimulation amplitudes within each polarity condition (one-way repeated-measures ANOVA followed by Tukey's multiple comparisons test; see Supplementary Statistical Tables). **F,** Peak amplitudes of Alb–mScarlet across stimulation amplitudes and polarities (two-way repeated-measures ANOVA followed by Bonferroni's multiple comparisons test; see Supplementary Statistical Tables). Data are presented as mean ± SEM (n = 4).

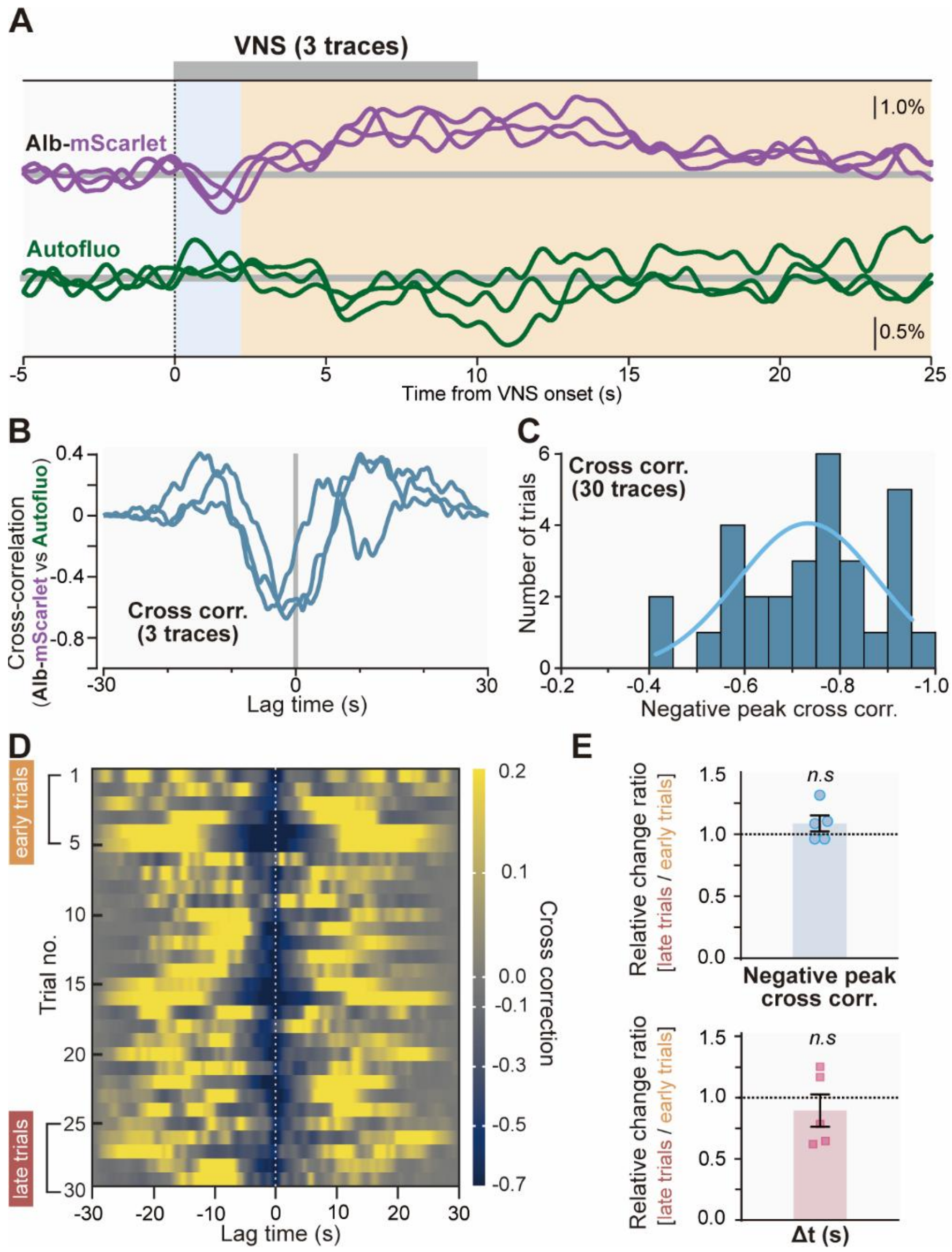

Figure S7: Cross-correlation between Alb–mScarlet and autofluorescence across VNS trials. **A,** Three representative simultaneous traces of Alb–mScarlet and autofluorescence. Colors indicate the two sequential temporal phases defined in Fig. 5**B**. **B,** Corresponding cross-correlation traces overlaid for the examples shown in **A**. **C,** Distribution of negative peak cross-correlation values across VNS series. A Gaussian fit is overlaid; normality was assessed using the Shapiro–Wilk test. **D,** Trial-by-trial cross-correlation analysis across a VNS series in an example mouse. Definitions of early and late trials are shown in Fig. S1**C**. **E,** Relative changes in negative peak cross-correlation and lag time between early and late trials (one-sample t-tests; negative peak: $t_{(4)} = 1.374$, $p = 0.24$, n.s.; lag time: $t_{(4)} = 0.782$, $p = 0.48$, n.s.). Data are presented as mean ± SEM (n = 5).

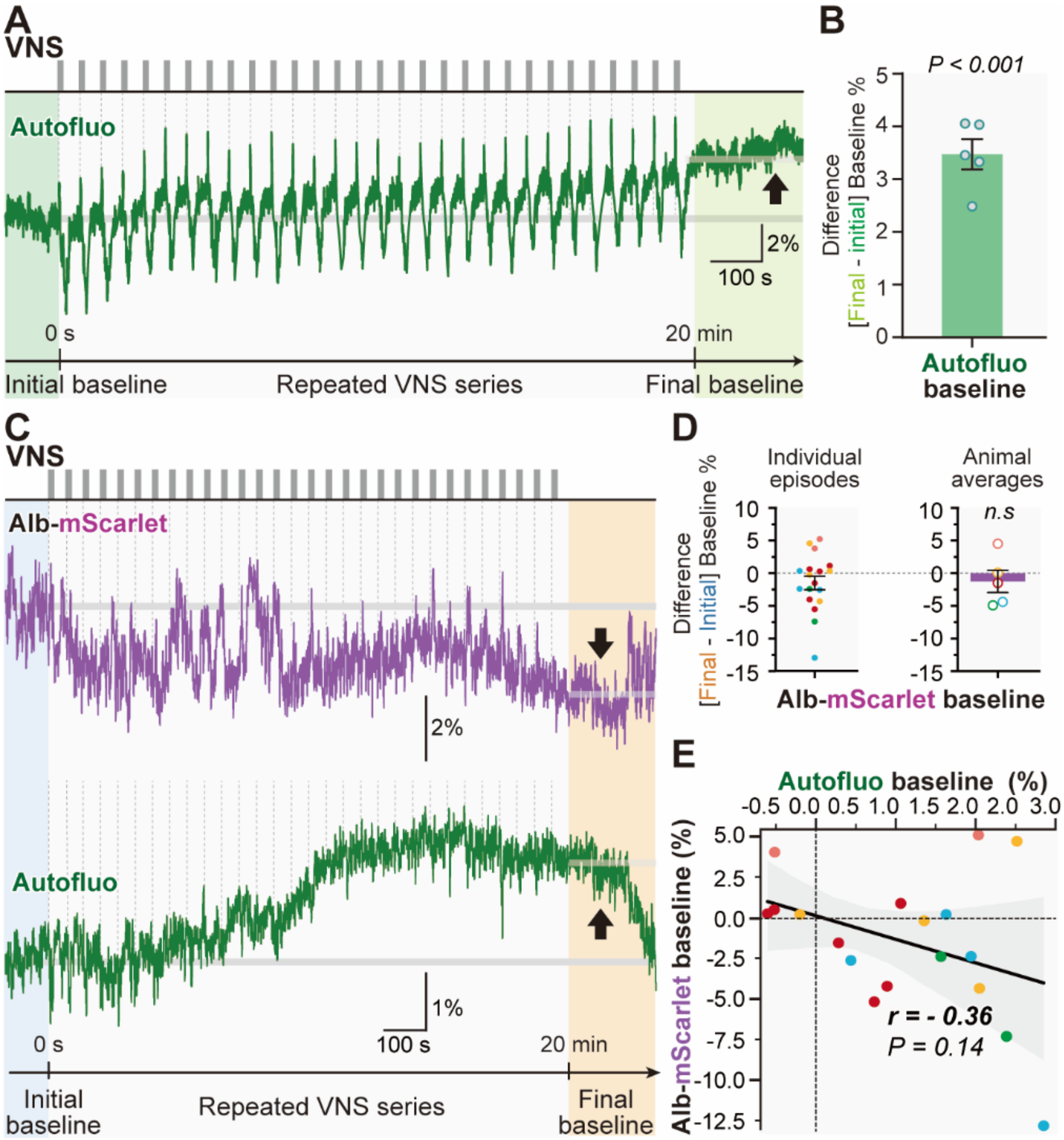

Figure S8: Baseline changes of autofluorescence and Alb–mScarlet across repeated VNS trials. **A,** Representative autofluorescence trace across repeated VNS trials. **B,** Autofluorescence baseline increased significantly following repeated VNS trials (one-sample t-test, $t_{(4)} = 12.10$, $p < 0.001$; n = 5). **C,** Representative simultaneous Alb–mScarlet and autofluorescence traces. **D,** Alb–mScarlet baseline levels at the episode level (left) and averaged within animals (right), showing no significant change (one-sample t-test, $t_{(4)} = 0.720$, $p = 0.51$, n.s.; n = 5). **E**, Relationship between Alb–mScarlet and autofluorescence baseline levels. No significant correlation was observed (r = −0.36, $p = 0.14$, n.s., linear regression), indicating a dissociation between their long-term baseline changes following repeated VNS. Each data point represents the average of 30 trials from a single VNS series (n = 18 episodes from 5 mice). Colors indicate individual animals. Data are presented as mean ± SEM.

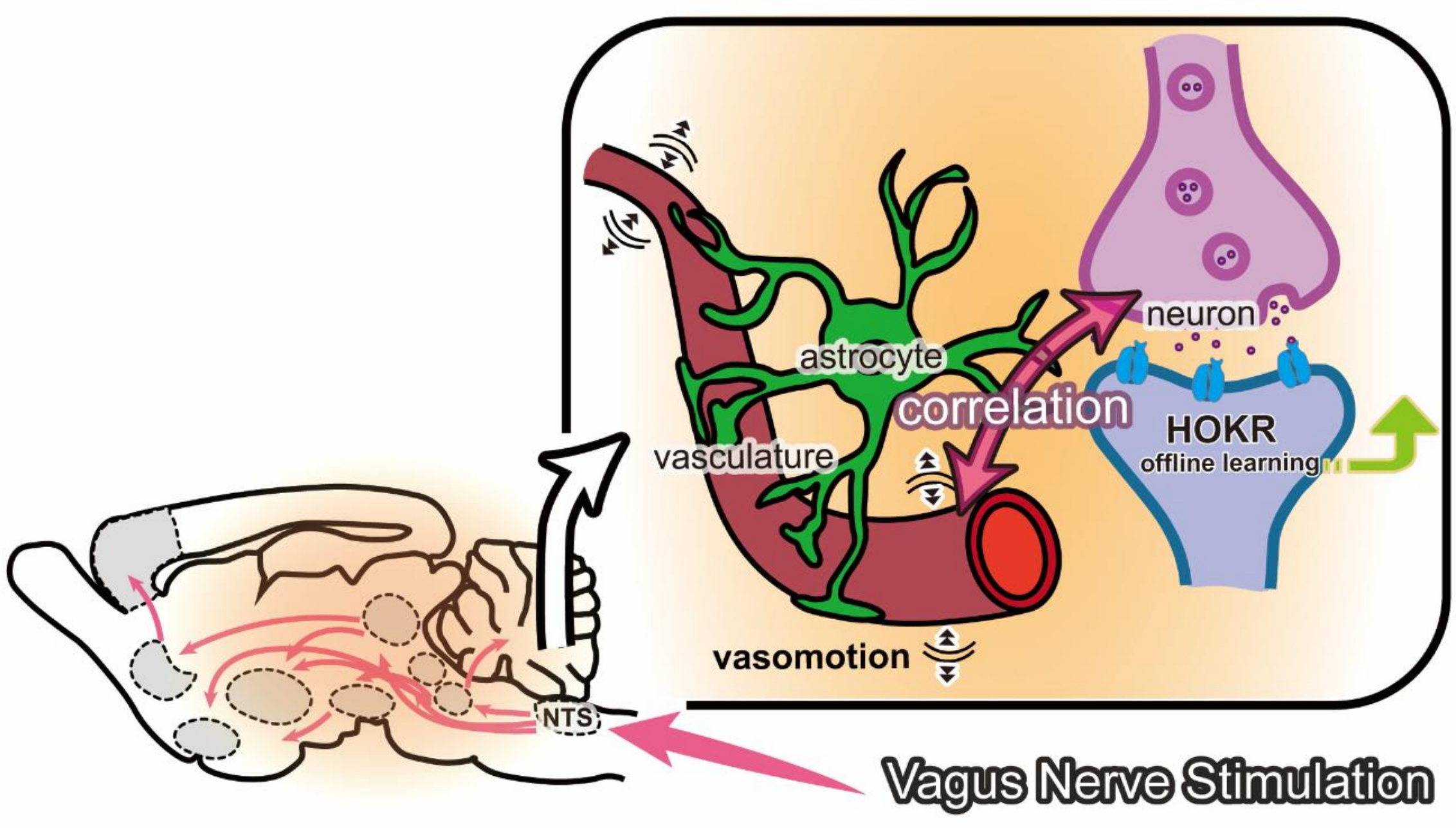

Figure S9: Conceptual model of vagus nerve stimulation–induced vascular and neurometabolic dynamics associated with long-term learning. Schematic illustration summarizing the proposed framework of this study. Vagus nerve stimulation (VNS) primarily activates afferent pathways projecting to the nucleus of the solitary tract (NTS), which in turn engage multiple central brain regions and promote the release of neuromodulatory transmitters. Through these pathways, VNS is associated with the induction of vascular volume fluctuations in the cerebellar flocculus. Such vasomotion may influence the local brain environment surrounding astrocytes and neurons. We further show that VNS delivered after horizontal optokinetic response (HOKR) training enhances multi-day long-term motor learning performance (late-offline learning). Although the relationship between vascular dynamics and learning remains correlational, these findings suggest a potential interaction between peripheral input, vascular activity, and neurometabolic processes. These processes may modulate the likelihood of plasticity, i.e., the metaplasticity of neuronal circuits. This framework highlights the possibility that peripheral inputs can influence central brain function by shaping vascular and metabolic states, thereby modulating the capacity for long-term learning.
